# DNA sequence-dependent formation of heterochromatin nanodomains

**DOI:** 10.1038/s41467-022-29360-y

**Published:** 2022-04-06

**Authors:** Graeme J. Thorn, Christopher T. Clarkson, Anne Rademacher, Hulkar Mamayusupova, Gunnar Schotta, Karsten Rippe, Vladimir B. Teif

**Affiliations:** 1 School of Life Sciences, University of Essex, Wivenhoe Park, Colchester, CO4 3SQ UK; 2 Division of Chromatin Networks, German Cancer Research Center (DKFZ) & Bioquant, Heidelberg, 69120 Germany; 3 Division of Molecular Biology, Biomedical Center (BMC), Faculty of Medicine, Ludwig-Maximilians-University (LMU) Munich, Großhaderner Straße 9, 82152 Martinsried, Germany; 4 Present Address: Barts Cancer Institute, Queen Mary University of London, Charterhouse Square, London, EC1M 6BQ UK; 5 Present Address: University College London, Gower St, Bloomsbury, London, WC1E 6BT UK

**Keywords:** Gene silencing, Epigenomics, Gene regulation, Histone post-translational modifications, Biological physics

## Abstract

The mammalian epigenome contains thousands of heterochromatin nanodomains (HNDs) marked by di- and trimethylation of histone H3 at lysine 9 (H3K9me2/3), which have a typical size of 3–10 nucleosomes. However, what governs HND location and extension is only partly understood. Here, we address this issue by introducing the chromatin hierarchical lattice framework (ChromHL) that predicts chromatin state patterns with single-nucleotide resolution. ChromHL is applied to analyse four HND types in mouse embryonic stem cells that are defined by histone methylases SUV39H1/2 or GLP, transcription factor ADNP or chromatin remodeller ATRX. We find that HND patterns can be computed from PAX3/9, ADNP and LINE1 sequence motifs as nucleation sites and boundaries that are determined by DNA sequence (e.g. CTCF binding sites), cooperative interactions between nucleosomes as well as nucleosome-HP1 interactions. Thus, ChromHL rationalizes how patterns of H3K9me2/3 are established and changed via the activity of protein factors in processes like cell differentiation.

## Abstract (web summary)

The ability to predict epigenetic regulation is an important challenge in biology. Here the authors describe heterochromatin nanodomains (HNDs) and compare four different types of H3K9me2/3-marked HNDs in mouse embryonic stem cells. They further develop a computational framework to predict genome-wide HND maps from DNA sequence and protein concentrations, at single-nucleotide resolution.

## Introduction

Cell type specific gene expression programs are established by distinct patterns of active and silenced chromatin states. One important type of a repressive heterochromatin state is characterized by di- or trimethylation of histone H3 lysine K9 (H3K9me2/3) and has heterochromatin protein 1 (HP1) as a marker^1,2^. It can comprise megabase-size regions that are found, for example, in pericentromeric regions of mouse and Drosophila. In addition, mammalian genomes are structured by tens of thousands of much smaller H3K9me2/3 heterochromatin loci with a typical size of 0.7–2 kb referred to here as heterochromatin nanodomains (HNDs)^3–8^. Their typical extension of around 3–10 nucleosomes is similar to the chromatin domain size determined by Micro-C^9^, corresponding to approximate dimensions of 40–70 nm^10^. In the present study, we investigate the mechanism by which four different types of HNDs form. HNDs have been identified in mouse embryonic stem cells (ESCs) and were found to be dependent on the following factors: (i) histone methyltransferases SUV39H1 (KMT1A) and SUV39H2 (KMT1B) referred to here as SUV39H that set H3K9me3 marks^3,4^; (ii) methyltransferase GLP (G9a like protein, KMT1D) that catalyses the formation of H3K9me2^5^; (iii) transcription factor ADNP that recruits the chromatin remodeller CHD4 as well as HP1β/γ for H3K9me3 mediated gene silencing^6^; (iv) chromatin remodeller ATRX that induces the formation of H3K9me3 HNDs at repeat sequences^7^.

Various biophysical models have been developed to describe the molecular mechanisms that govern the formation of chromatin domains with specific histone modifications^11–24^. These models typically include DNA-protein binding and enzymatic reactions to account for epigenetic phenomena like the establishment of bistable states. In such models, the spreading of a given modification to adjacent nucleosomes on the chain is explained by nearest-neighbour feedback mechanisms and/or long-range interactions, e.g., through looping of the nucleosome chain. In addition, several mechanisms have been proposed to explain what stops heterochromatin spreading and sets domain boundaries: (i) The dynamic properties of the nucleosome chain can inherently limit the interactions of a nucleosome that could propagate H3K9me3 modifications in the presence of counteracting enzymatic activities that remove this mark^24^. (ii) A certain H3K9me3 density threshold may be required for effective chromatin-association of the histone methyltransferase that sets the modification^25^. (iii) An island of nucleosomes marked by phosphorylation of histone H3 at serine 10 ^26^, nucleosome-depleted regions^27^ or DNA-bound molecules such as RNA polymerase or CTCF^28,29^ could act as boundary elements that interfere with the nearest-neighbour type spreading of histone modifications.

Interestingly, ectopic HNDs can be induced by artificially tethering the H3K9 methylase Clr4 in yeast^30^ or HP1 in mouse ESCs^14^ to chromatin. These findings suggest that the DNA-sequence-directed binding of protein factors could serve to nucleate the formation of endogenous HNDs. However, the theoretical models mentioned above lack the ability to integrate DNA sequence-directed binding with the formation of chromatin domains that are defined by nucleosomes with different histone modifications on a genome-wide scale. Furthermore, it is an open question if HNDs are inherently limited in size as proposed for ectopic HP1 induced domains^14,15^ or if specific mechanisms determine their boundaries. These issues are addressed here by introducing the Chromatin Hierarchical Lattice (ChromHL) framework. ChromHL uses the transfer matrix formalism of statistical mechanics to calculate the DNA sequence-specific equilibrium occupancy of transcription factors (TFs) and other chromatin proteins along the genome at single-nucleotide resolution^17,31^ and links it to nucleosome states with distinct modifications. This approach allows us to describe HND formation as a general mechanism involving DNA sequence-specific binding of nucleation factors and formation of HND boundaries that are determined mainly either by the DNA sequence or by nucleosome-nucleosome/HP1 interactions. By fitting the ChromHL predicted genome-wide HND patterns to the experimental data, crucial DNA sequence and chromatin features are revealed that determine the localization and extension of H3K9me2/3 HNDs throughout the genome.

## Results

### Four types of endogenous HNDs are distinguished in ESCs

**Fig. 1:**
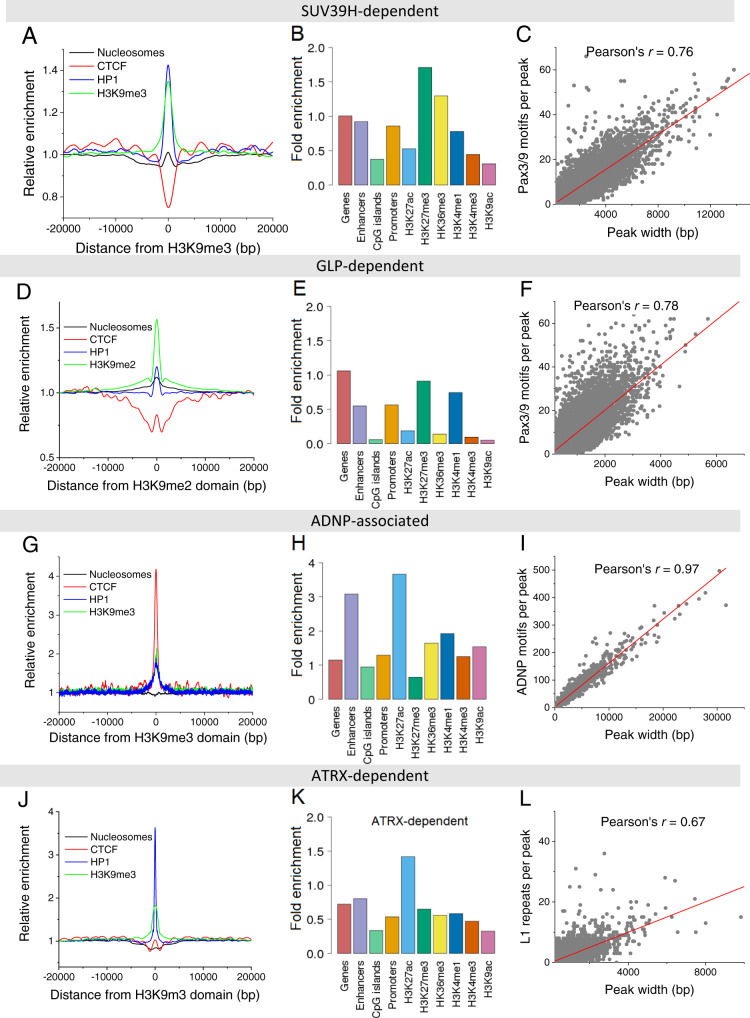
Average profiles and enrichments of chromatin features across four different endogenous HND types. Left, nucleosome occupancy and density of CTCF, HP1 and H3K9me3 (or H3K9me2 for GLP-dependent peaks) Middle, relative enrichment of different features. Right, correlation between peak width and the number of heterochromatin-initiating motifs per peak. **A**–**C** SUV39H-dependent HNDs (*n* = 36,764). **D**–**F** GLP-dependent HNDs (*n* = 48,881). **G**–**I** ADNP-associated HNDs (*n* = 4673). **J**–**L** ATRX-dependent HNDs (*n* = 13,113).

We first compared the structure and composition of four types of HNDs marked by H3K9me2/3 in ESCs that were dependent on SUV39H^3,4^ and GLP^5^ or marked by ADNP^6^. In addition, a dataset for ATRX-dependent H3K9me3 HNDs was generated here by ChIP-seq of wild-type (WT) ESCs and *Atrx* knock out (KO) cells (Supplementary Fig. 1). For SUV39H, GLP and ATRX HNDs, we called H3K9me2/3 ChIP-seq peaks separately in WT and KO conditions. Subsets of peaks were identified that were present in WT but absent in the KO cells. This yielded 36,764 (*Suv39h1*/*Suv39h2* KO, H3K9me3), 48,881 (*Glp* KO, H3K9me2) and 13,113 (*Atrx* KO, H3K9me3) regions that change their H3K9 methylation state upon the knockout of the indicated protein factor in ESCs. In the case of the ADNP dataset, 4673 H3K9me3 domains were called by intersecting H3K9me3 domains with regions bound by ADNP in wild-type ESCs. Next, we calculated average profiles of HP1α, CTCF, nucleosome density and H3K9 methylation as a function of the distance from the centres of SUV39H-, GLP-, ADNP- and ATRX-associated HNDs (Fig. 1). In addition, the corresponding profiles for H3K27me3, H3K4me1, CpG methylation, different chromatin states defined by combinatorial histone marks from the analysis with ChromHMM and the read mappability were computed (Supplementary Figs. 2, 3). In all cases, HP1 binding and H3K9me3 (or H3K9me2 in the case of GLP-dependent regions) were enriched at the peak centre. In SUV39H-, GLP- and ATRX-dependent HNDs, CTCF was found to be depleted while the nucleosome density was increased. In contrast, CTCF was significantly enriched in ADNP-associated HNDs that also showed a slight nucleosome density reduction. Interestingly, ADNP-associated HNDs overlapped with enhancers and were enriched in active histone marks such as H3K27ac, H3K4me1 and H3K36me3. Thus, this type of H3K9me3 nanodomains is likely to have functional roles different from that of the canonical silenced heterochromatin state that lacks these active histone marks. DNA methylation was strongly enriched in SUV39H-dependent regions but not in the other three types of HNDs. An interesting feature of GLP-dependent heterochromatin was the presence of repetitive regions as apparent from the drop in the mappability index. Thus, all four types of nanodomains studied here had distinct chromatin features.

### Recurring sequence motifs can act as HND nucleation sites

For SUV39H-dependent H3K9me3 nanodomains, we followed the hypothesis that heterochromatin nucleation is induced by binding sites of transcription factors PAX3 and PAX9^3^. We found that 92.4% of SUV39H-dependent HNDs indeed contained the sequence motif of the PAX3/9 binding site. The same motif was also detected in 95.9% of the GLP-dependent nanodomains, suggesting that it could also drive the formation of this nanodomain type. Interestingly, the sizes of both SUV39H- and GLP-dependent HNDs correlated well with the number of PAX3/9 motifs per corresponding HND (Pearson’s *r* = 0.76 and 0.78, correspondingly; Fig. 1C, F). For ADNP, we derived the position weight matrix (PWM) from the ChIP-seq data^6^ (see Methods) and used it to correlate domain extension with the number of ADNP binding motifs. This resulted in Pearson’s *r* = 0.97 for the HNDs defined from the intersection of H3K9me3 and ANDP ChIP-seq peaks (Fig. 1I), which means that the number of ADNP motifs per HND is a very good predictor of the HND size. Nucleation of heterochromatin formation by ATRX involves the recruitment of SETDB1 and/or SUV39H1 that set the H3K9me3 modification but different targeting mechanisms for these enzymes have been proposed^32–35^. Accordingly, we evaluated DNA sequence motifs that could act as nucleation sites at the 13,113 ATRX-dependent H3K9me3 nanodomains that we identified. (i) Only 4,851 (37%) of these regions contained PAX3/9 motifs. (ii) The telomeric repeat-containing RNA (TERRA) has been reported to compete with ATRX binding at the telomeric repeat sequence TTAGGG interspersed in the genome^35^. We found this sequence in 5,599 ATRX HNDs (43%). (iii) ATRX is known to bind to G-quadruplexes^34,36^. Accordingly, we searched for G-quadruplex motifs^37^ within HNDs. However, only 1026 ATRX HNDs (8%) contained such motifs (Supplementary Fig. 10). (iv) IAP repeats containing a 160 bp sequence motif termed SHIN sequences have been previously reported to initiate ATRX heterochromatin^32^. However, the SHIN sequence was absent in ATRX-dependent nanodomains and <1% of ATRX HNDs intersected with annotated IAP repeats based on UCSC RepeatMasker. (v) We analysed other repeat sequences and found that all ATRX-dependent regions contained at least one LINE1 sub-repeat of 200–300 bases (Supplementary Table 1). Pearson’s correlation between the size of ATRX-dependent HNDs and the number of L1 occurrences per HND reached *r* = 0.67 (Fig. 1L). Thus, for all four different types of HND we identified DNA sequence motifs that could act as nucleation site (Supplementary Table 2).

### ChromHL predicts HNDs from a DNA sequence-informed hierarchical lattice model

**Fig. 2:**
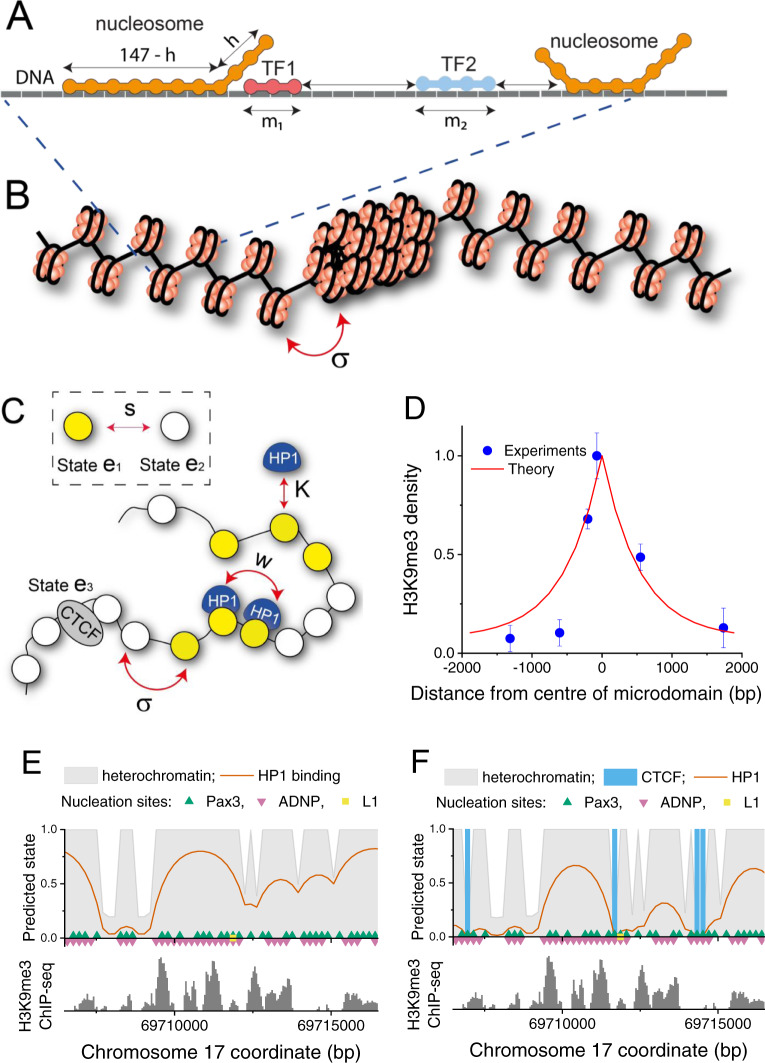
ChromHL framework for HND description. **A** A lattice model with single-base pair resolution for TF binding to DNA in the context of nucleosomes that can partially unwrap. **B** Schematic representation of the nucleosome array containing two types of packing. The boundary between different chromatin states is characterised by the statistical weight parameter σ. It could reflect that inter-nucleosome interactions in different packing states are different and thus create a boundary between them. **C** Description of chromatin in ChromHL. Chromatin is described by a lattice of nucleosome units. These lattice units can exist in different states. In the present study, three states *e*_1_, *e*_2_ and *e*_3_ exist that correspond to a H3K9me2/3 modified nucleosome, an unmodified nucleosome or a unit without a nucleosome but with CTCF bound. Lattice units can switch between different states with probability *s*(*e*_1_, *e*_2_). Chromatin proteins can shift this equilibrium by binding nucleosomes with different binding constants *K*(*e,g*) depending on the protein type *g* and the chromatin state *e* of a given lattice unit while interactions between proteins bound to neighbouring lattice units is described by the cooperativity parameter *w*. **D** H3K9me3 profile predicted by ChromHL in comparison to experimentally determined data in mouse embryonic fibroblasts for an ectopic HND^14^, with best fit parameters *s* = 7.1e−3, σ = 1.16e−5, *w* = 8.25e3. The mean occupancy (normalised so the maximum occupancy is 1) is shown, along with bars showing standard deviation from two biological replicates. **E** Model predictions (top panels) and experimental ChIP-seq profiles of endogenous H3K9me3 (bottom panels) for an example genomic region in ESCs. This model includes HND nucleation at PAX3/9, ADNP and L1 sequence motifs, but does not consider CTCF binding. In this calculation σ = 1, and therefore the main determinants of HNDs are the nucleation sites in the DNA sequence. **F** Same as panel **E** but including CTCF binding as a factor that determines HND boundaries. Including CTCF improves the agreement of the predicted HND profile with the experimental data.

Next, we set out to model the genomic distributions of all four different HND types based on nucleation at the specific DNA-sequence motifs described above and evaluating the mechanisms that determine the domain boundaries. To accomplish this task, we developed the hierarchical lattice framework ChromHL (Fig. 2). It is based on a DNA-binding lattice model at single-nucleotide resolution as its first hierarchy level to determine the arrangement of nanodomain-nucleating proteins such as PAX3/9, ADNP or 3D-organising factors such as CTCF. On top of the DNA sequence-based lattice model, a lattice model with nucleosome-size units is defined to describe nucleosomes in different states that are characterized by their histone modifications and bound proteins such as HP1. In addition, ChromHL accounts for nucleosome-nucleosome interactions that depend on the nucleosome state. The maps of chromatin nanodomains and bound proteins are calculated with the transfer matrix formalism of statistical mechanics (Methods, Supplementary Information, Supplementary Fig. 18). Partial unwrapping of DNA from the nucleosome core at single-nucleotide resolution as well as competitive and cooperative DNA binding of different protein species was implemented according to the approach described previously^38,39^. Furthermore, a lattice unit *n* was allowed to be in three different chromatin states *e*(n) in addition to the bound/unbound protein states (Fig. 2B, C): (i) a nucleosome with unmethylated H3K9 tails, (ii) a nucleosome with methylated H3K9 tails, and (iii) bound CTCF while a nucleosome is missing from the lattice unit. This model can be extended to include other nucleosome states as needed. The main input parameters for the ChromHL calculations are (i) the DNA sequence, (ii) weight matrices of size 4 x *m* setting DNA binding affinities for each protein type *g* which covers *m*(*g*) bp upon binding, (iii) binding constants *K*(*n*,*g*) for protein binding to a nucleosome in dependence of its state, (iv) cooperativity parameters *w*(*g*_1_, *g*_2_, *l*) between protein types *g*_1_ and *g*_2_ separated by *l* lattice units, (v) protein concentrations *c*(*g*), (vi) the statistical weight *s*(*e*_1_, *e*_2_) for a transition of a lattice unit between chromatin states *e*_1_ and *e*_2_, and (vii) the nucleosome-nucleosome interaction potential σ(*e*_1_, *e*_2_) (Fig. 2A). The latter interaction potential σ(*e*_1_, *e*_2_) depends on the states of the interacting nucleosomes, *e*_1_ = 1 and *e*_2_ = 2. Thus, if neighbouring clutches of nucleosomes belong to different states, a difference in nucleosome-nucleosome interactions between these two states can create “surface tension” at the boundary with additional energy costs. Another important feature of the model is the contribution of nucleosome-binding proteins such as HP1, which is considered here in the context of HNDs. HP1 binds stronger to nucleosomes that are in the methylated H3K9 state, which shifts the thermodynamic equilibrium towards heterochromatin formation for the regions where it is bound. Furthermore, neighbouring HP1 molecules bind cooperatively as described by the parameter *w*, which can contribute to heterochromatin spreading beyond nucleation motifs.

### An ectopically induced HND is confined by boundary interactions of nucleosomes

We first applied the ChromHL model to an artificial system of a single HND with a well-defined nucleation point and no sequence-defined boundaries. Such an ectopic HND was created in experiments of Hathaway et al. by tethering HP1α to the *Oct4* locus and inducing local H3K9me3 enrichment^14^. The experimentally determined H3K9me3 profiles decay to zero at distances of ~2 kb from the initiation site. We performed parameter optimisation with ChromHL to match this experimental H3K9me3 profile (Fig. 2D, Supplementary Fig. 4). The heterochromatin spreading for this system is determined by three parameters: (i) The binding affinity of HP1 to H3K9me3-modified nucleosomes, which was estimated to be 10-fold stronger than that to unmodified H3K9 following the previous analysis^18^. (ii) The contact cooperativity value *w* for HP1-HP1 interaction has not been well defined in the previous studies^18,40,41^. The best fit of the model to the H3K9me3 profile reported by Hathaway et al. returned value of *w* = 4060. It is indicative of a significant positive binding cooperativity as compared to *w* = 1, which would represent the independent binding of HP1 to adjacent nucleosomes. (iii) The parameter σ describes the “energetic boundary” between neighbouring chromatin packing types *e*_1_ and *e*_2_. A value of σ = 1 would mean that this transition is not associated with energetic costs. However, for the ectopic HND our best fit required σ ~10^−5^. The magnitude of this σ-value is characteristic for highly cooperative transitions such as, for example, DNA melting^42^. The low value of σ ~10^−5^ reflects that the domains become intrinsically confined without additional DNA sequence-dependent contributions. This behaviour is different from endogenous HNDs considered below.

### ChromHL predicts experimental maps of endogenous HNDs in living cells

The nucleation sites of endogenous HNDs are determined by the genomic location of PAX3/9, ADNP and L1 sequence motifs derived above. When combined, they allowed a good match between computationally predicted and experimental HND profiles as shown for an exemplary region in ESCs (Fig. 2E, F). In addition, accounting for CTCF binding led to an even better agreement of theory and experiment (Fig. 2F) as compared to the model without CTCF (Fig. 2E, Supplementary Fig. 5). Thus, endogenous HNDs depend to a larger degree on the DNA sequence than the ectopic HND (Fig. 2D). On the other hand, adding strong nucleosome-nucleosome interactions with σ ~10^−5^ as in the ectopic example led to merging of neighbouring endogenous HNDs, while the fine structure of the H3K9me3 profile was lost (Supplementary Figs. 5, 7). In the case of the endogenous SUV39H HNDs, a better fit was obtained with σ ~1. This means that the energy of nucleosome-nucleosome interactions at HND boundary did not exhibit any abrupt change, and CTCF binding was the main determinant of boundary formation. This model results in a larger number of smaller HNDs as the DNA sequence introduces many additional constraints to HND sizes (Supplementary Figs. 5, 7). Thus, ChromHL allows us to separate different contributions of genetic and epigenetic interactions to the domain boundaries.

### Average endogenous nanodomain profiles have a typical extension of 0.7–2 kb

**Fig. 3:**
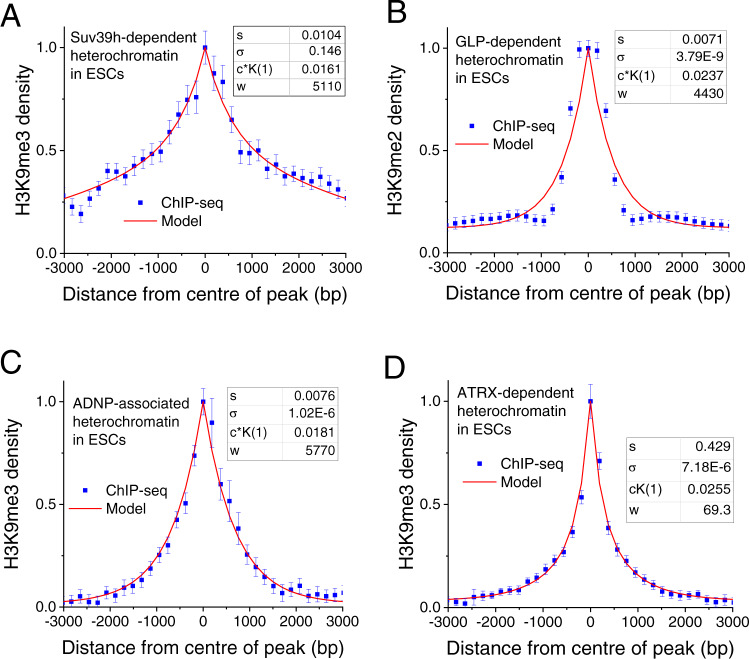
Shape of averaged H3K9me2/3 profiles for different HND types. The experimental H3K9me2/3 ChIP-seq densities were normalised to [0, 1]. Parameters: *s* is the statistical weight for the nucleosome conversion to heterochromatin state, σ =  σ(1, 2) is the weight for interaction between nucleosomes in states 1 (unmethylated) and 2 (methylated). *c* × *K*(1) is the product of the local concentration of HP1 proteins in state 1 and the binding constant of HP1 for this state. The mean and standard deviations from two replicates per experiment are shown. **A** SUV39H-dependent HNDs. **B** GLP-dependent HNDs. **C** ADNP-associated HNDs. **D** ATRX-dependent HNDs.

The characteristic aggregated H3K9me3 profiles of SUV39H-, GLP- ADNP- and ATRX-dependent HNDs in ESCs are shown in Fig. 3. These experimental profiles were obtained by averaging all individual regions with the corresponding heterochromatin subtypes centred at the summits of ChIP-seq peaks (H3K9me3 in the case of SUV39H, ADNP and ATRX, and H3K9me2 in the case of GLP). The resulting profiles resemble that of the ectopically induced H3K9me3 domain (Fig. 2D). Accordingly, the computational analysis of the aggregated data with the ChromHL model yielded a good fit to the same model that was used for the ectopic HND: a central nucleation site and self-contained extension due to an unfavourable chromatin state transition as reflected by a low σ value (Fig. 3, Supplementary Fig. 4). However, a closer inspection of the data revealed a significant variation of σ with a relatively high value of σ = 0.14 retrieved for SUV39H HNDs. Importantly, the information about molecular mechanisms that define the boundaries for individual regions was lost in the aggregated plots. Therefore, in the next part of this study, we performed genome-wide analysis of individual domains.

### DNA sequence is a major determinant of endogenous HNDs

**Fig. 4:**
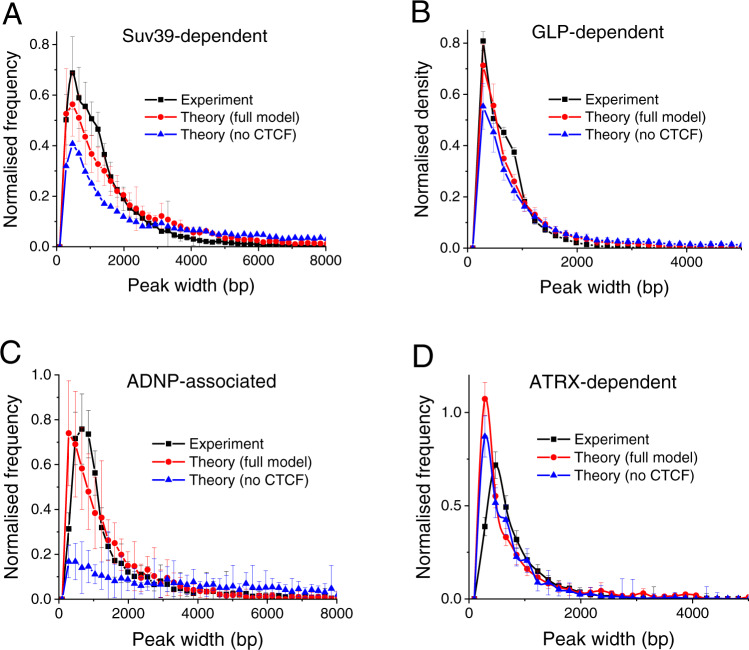
ChromHL predictions compared with the experimental distribution of HND sizes. The experimental nanodomain sizes given by H3K9-methylated ChIP-seq peaks (black) are plotted against the theoretically predicted ones calculated with ChromHL. The full model (red) and the simplified model without CTCF (blue) are shown. The mean and standard deviations of the bar heights of the histogram are shown, averaged over chromosomes 1-19 and X. Detailed best fit parameters are given in Supplementary Table 3. **A** SUV39H-dependent HNDs. **B** GLP-dependent HNDs. **C** ADNP-associated HNDs. **D** ATRX-dependent HNDs computed with the L1 repeats-based model. *σ* = 1 for SUV39H-, GLP- and ADNP-HNDs, and *σ* = 0.01 for ATRX-HNDs.

Next, we investigated the effect of DNA sequence on heterochromatin initiation and localisation. A genome-wide analysis was conducted for the four different HND types with the nucleation sequence motifs derived above. In our analysis we considered both the effect of CTCF and cooperative HP1 binding to neighbouring nucleosomes with a 10-fold increase of the binding constant at H3K9me2/3-modified nucleosomes (Fig. 4, Supplementary Table 3). The comparison of predicted and experimentally determined distributions of SUV39H-dependent HND sizes showed a significant improvement if CTCF binding was included and yielded a fit value of σ = 1 (Fig. 4A, B, Supplementary Figs. 6, 8, 9). Interestingly, the model derived for SUV39H-HNDs was also well suited to describe the extension of GLP-HNDs marked by H3K9me2 (Fig. 4B, Supplementary Fig. 9), although protein binding to PAX3/9 motifs differs between the two. For ADNP-HNDs we used the PWM derived from ADNP ChIP-seq to define ADNP binding sites for domain nucleation (Fig. 4C). Again, including CTCF binding improved the model with σ = 1. For ATRX-HNDs, the centres of the L1 sub-repeats L1Md_F2, L1Md_T, L1Md_A and L1Md_F (of approximately 200–300 bp width) were used as nucleation sites. The resulting model describes the experimental data well (Fig. 4D, Supplementary Fig. 11). Notably, and in contrast to the three other heterochromatin types, the effect of CTCF was negligible. Furthermore, the best fit value of the boundary weight yielded σ = 0.01, corresponding to a free-energy change ≈ 4.6 kT per boundary. This energy is comparable to typical nucleosome-nucleosome interactions^18^ and very different from the value of σ = 1 (energy change ≈ 0 kT) obtained as best fit for the other heterochromatin types. Thus, we conclude that boundaries of ATRX-HNDs are determined mostly by unfavourable transitions to the flanking chromatin states. In contrast, the SUV39H-, GLP- and ADNP-HNDs were described best with the same model that included sequence-specific binding of TFs (PAX3/9, ADNP) as a nucleation site, CTCF binding sites as boundary elements and a value of σ  = 1 indicative of no energy penalty to the flanking chromatin states.

### HNDs differ in their nucleosome packing patterns

**Fig. 5:**
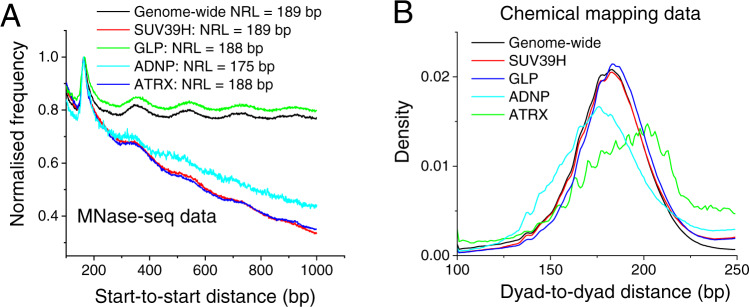
Nucleosome packing characteristics in different HND types. **A** Nucleosome start-to-start genome-wide distance distribution (black line) based on MNase-seq^54^ in SUV39H- (red), GLP- (green), ADNP- (light blue) and ATRX-HNDs (dark blue). **B** Nucleosome dyad-to-dyad distance distribution for nearest-neighbour nucleosomes based on chemical mapping^44^.

We further dissected the differences between SUV39H-, GLP-, ADNP- and ATRX-HNDs by assessing the nucleosome repeat length (NRL) inside these regions. We calculated average NRLs using MNase-seq data based on cutting DNA between nucleosomes^43^ (Fig. 5A) as well as the dyad-to-dyad frequency distribution using chemical mapping data based on cutting DNA at the nucleosome dyads^44^ (Fig. 5B). MNase-seq derived NRLs for SUV39H-, GLP-, and ATRX-dependent nanodomains of 188 or 189 bp were similar to the genome-wide NRL of 189 ± 1 bp. In addition, heterochromatin states defined previously based on H3K27me3 enrichment in ESCs using ChromHMM^45^ had a similar NRL value (Supplementary Fig. 14A). In contrast, ADNP-associated HNDs were characterised by a smaller NRL of 175 ± 1 bp. When considering the distribution of nucleosome dyad-to-dyad distances obtained from chemical cleavage at nucleosome dyads^44^ (Fig. 5B), SUV39H- and GLP-dependent heterochromatins again showed the same distribution as genome-average. In contrast, ATRX- and ADNP-HNDs clearly displayed a different distribution of dyad-to-dyad distances (Fig. 5B). Unlike NRL analysis based on MNase-seq, chemical mapping suggested that ADNP-HNDs had significantly smaller dyad-to-dyad distances, while the distribution of dyad-dyad distances in ATRX-HNDs was shifted to larger values in comparison to the genome average. Interestingly, in SUV39H- and GLP-HNDs, the distribution of dyad-to-dyad distances resembled that of H3K27me-enriched heterochromatin (Supplementary Fig. 14A). This is consistent with the fact that ~75% of SUV39H- and ~90% of GLP-dependent HNDs reside within H3K27me-enriched chromatin states. In contrast, only about 5% of ATRX-dependent HNDs were located within H3K27me3-enriched states^45^ (Supplementary Fig. 14B). We also studied the effect of different sizes of the ChromHL lattice unit on our predictions of the distribution of HND sizes, considered effective NRLs ranging from 161 to 199 bp. No significant effects of the NRL change on the nanodomain sizes per se were observed (Supplementary Fig. 13). This suggests that the differences in nucleosome packing found above may affect HND formation indirectly, e.g., by modulating the value of σ.

### HND redistribution during cell transition can be regulated by protein binding activity

**Fig. 6:**
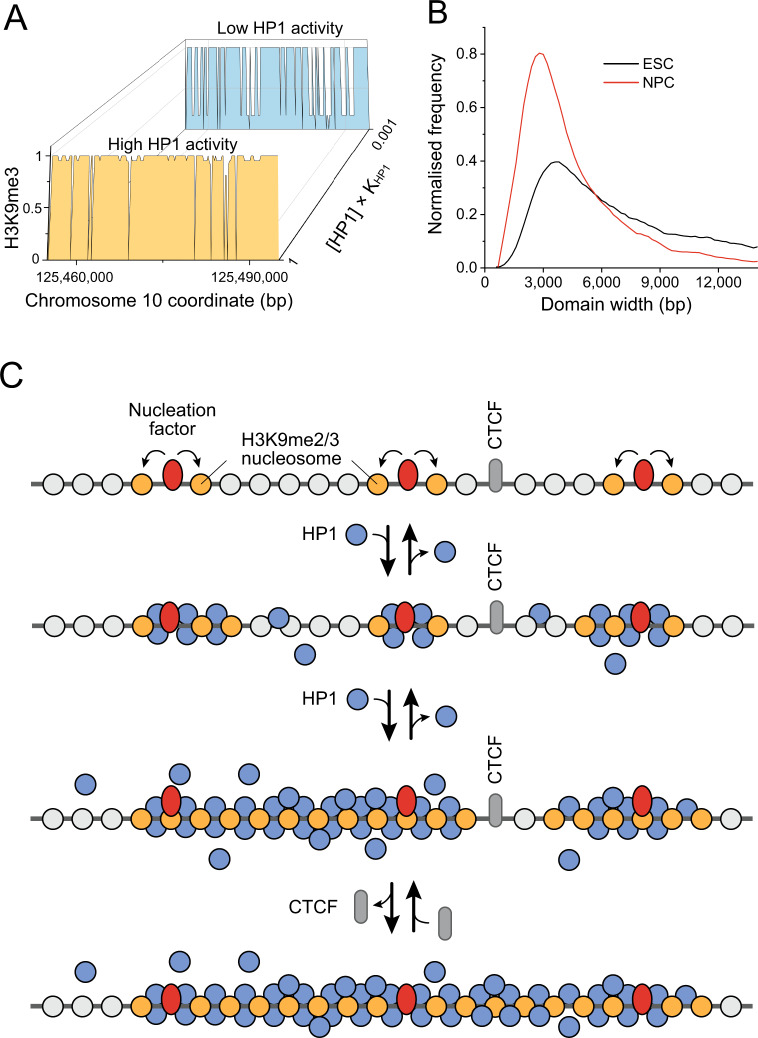
Dynamics of heterochromatin nanodomains during cell transitions. **A** Model-predicted heterochromatin state probability for the region centred on chr10:125454974-125464974, for large ([HP1] × *K*(HP1) = 1) and low concentration of free HP1 molecules ([HP1] × *K*(HP1) = 0.001). The model predicts that increasing concentration of free HP1 molecules leads to formation of larger heterochromatin domains. **B** Experimental distributions of H3K9me3 domains in ESCs (black) and NPCs differentiated from them (red). **C** Schematic model of factors that govern HND features. Heterochromatin nucleation sites are determined by the DNA sequence and concentration of molecules binding these sequences such as Pax3/9 and ADNP. Increasing the HP1 activity (the local concentration of free HP1 molecules) may lead to the broadening of heterochromatin domains, as observed during differentiation of ESC into NPCs^58^. In addition, CTCF binding site occupancy can affect heterochromatin domains boundaries.

H3K9me2/3 marks constitutive heterochromatin loci as well as cell type-specific regions that change during differentiation^46^. Furthermore, aberrant gain or loss of H3K9me3 is a feature of many cancers^47^. In our framework, the location and extension of HNDs is regulated by binding of PAX3/9, ADNP and CTCF. In general, the binding activity of these and other TFs can be regulated via their expression levels, subcellular localization and/or posttranslational TF modifications^31^. In addition, the activity of H3K9-modifying enzymes (SUV39H1/2, GLP, SETDB1) or H3K9me2/3-binding proteins like HP1 could determine cell-type-specific HND patterns. Significant changes in the binding properties and genomic localization of HP1 molecules occur during cell differentiation^48^. Accordingly, we explored the effect of the change of HP1 concentration on the structure for SUV39H-dependent H3K9me3 HNDs (Fig. 6A). Our model predicts that decreasing the concentration of free HP1 molecules reduces HND size. Consistent with this prediction, the experimental distributions of SUV39H-dependent HNDs in ESCs vs neural progenitor cells (NPCs) generated by in vitro differentiation show a significant decrease of average HND size (Fig. 6B). A similar effect takes place for all four types of HNDs upon differentiation of ESCs to NPCs (Supplementary Fig. 15). HND shrinking/expansion in dependence of HP1 activity can be further modulated by differential binding of HND-initiating TFs and CTCF as was shown above (Fig. 2E, F). Thus, the formation of HNDs is partially hard-wired in the DNA sequence but their cell-type-specific patterns are dependent on the activities of additional factors (Fig. 6C).

## Discussion

We conducted a systematic comparison of four types of HNDs in ESCs and quantitatively described them with ChromHL, a framework allowing sequence-specific prediction of HND maps. The SUV39H- and GLP-HNDs were well-described by a common model based on PAX3/PAX9-nucleation sites (Fig. 4A). The binding of PAX3/9 has been previously suggested to target SUV39H-dependent H3K9me3 domains^3,4^. Our analysis supports this conclusion as we find a high correlation between the number of PAX3/9 motifs per domain and domain size (Fig. 1C, F, Supplementary Table 2). In addition, the locations of domain boundaries can be rationalized by CTCF binding. Interestingly, while a similar model of sequence-dependent HND extension worked well for GLP-dependent HNDs that carried the H3K9me2 mark, these two types of HNDs are largely not overlapping (Supplementary Fig. 19). This means that for SUV39H- and GLP-HNDs we can predict the extensions of the corresponding H3K9me3/2 HNDs from the binding profiles of PAX3/9 and CTCF (Fig. 2E, F). To independently define their location, additional factors would have to be included that determine the differential use of PAX3/9 nucleation sites for these two types of HNDs. The ADNP-associated HNDs also displayed a very good fit to this type of ChromHL model with the nucleation site defined by the ADNP motif computed here (Fig. 4C, Supplementary Table 2). It is noted that the relation of DNA sequence and HND nucleation site could extend the targeting of a single factor as multiple TFs might be involved. This consideration is particularly relevant for the case of ATRX-dependent HNDs. Our analysis identified L1 repeat family as the primary sequence feature responsible for HND nucleation (Fig. 1L). However, the correlation of the sizes of ATRX-HNDs with the number of the L1 sub-repeats (L1Md_F2, L1Md_T, L1Md_A and L1Md_F) motifs per HND was only 0.67. Thus, the use of this sequence feature to model ATRX nanodomain formation with ChromHL resulted in a less good fit than the one obtained with the other three HNDs (Fig. 4D). We conclude that additional protein factors and nucleation mechanisms are likely at play for ATRX-dependent HNDs. It is also worth noting that ATRX knockout reduces H3K9me3 on IAPs but H3K9me3 is not lost entirely. Accordingly, these regions would not qualify as fully ATRX-dependent HNDs in the above analysis although ATRX affects H3K9me3 levels within these regions.

Our ChromHL analysis predicts that CTCF binding motifs at boundaries represent a major defining feature for the extension of SUV39H-, GLP- and ADNP-HNDs. This contribution of CTCF is consistent with our previous report where CTCF sites acted as bifurcation points for differential DNA methylation spreading upon TET1/2 knockout^29^. The contribution of CTCF to the spreading of H3K9 methylation arises from the current analysis. CTCF is known to be involved in the formation of topologically associated domains and loops, with the size of CTCF-demarcated loops/and in the range from 10 kb to 1 Mb^49^. In contrast, the nanodomains studied here are typically 0.7–2 kb in size. They involve weak CTCF binding sites. These sites are frequently not called with the typical peak detection thresholds used in the analysis of CTCF ChIP-seq data, which retrieve ~60,000 relatively strong binding sites. However, we propose here that weak CTCF sites are functionally important and define the H3K9me2/3 nanodomain structure in the genome by transient binding of CTCF, possibly in conjunction with other proteins. In our recent work, we reported that such CTCF motifs are enriched in DNA sequence repeats at sites of reduced nucleosome density^27^. Thus, these motifs may also be related to the loss of a nucleosome, which could affect the interactions between neighbouring nucleosomes at the boundary.

ChromHL modelling allowed us to uncouple DNA sequence determinants from thermodynamic constraints that limit the sizes of HNDs. The parameter σ defines the energetic cost of formation of a new boundary between chromatin states in analogy to the cooperativity constant used in statistical mechanical models that describe melting of the DNA double helix^42^. The associated boundary energies can, for example, arise from (un)favourable nucleosome stacking interactions between nucleosomes^50^. In the case of ectopic HNDs established in the experiments of Hathaway et al.^14^, the best fit of our model returned small σ values suggesting unfavourable nucleosome interaction energies at the domain boundaries (Fig. 2D). Similarly, small values were obtained when considering a hypothetic genomic region with the same H3K9 methylation profile as the one averaged over the individual HNDs (Fig. 3). However, such average profiles do not represent well the individual HNDs that have been used for the genome-wide calculation (Fig. 4). The fit that captured the distribution of SUV39H-, GLP- and ADNP-HNDs best was obtained with a boundary formation weight of σ = 1, indicating a lack of structural transitions from the H3K9me2/3 states. This finding suggests that the SUV39H-, GLP- and ADNP-dependent nanodomain size is mostly DNA sequence-determined by the respective nucleating TFs and CTCF. In contrast, ATRX-dependent H3K9me3 nanodomains did not fit to this model. A value of σ = 0.01 was retrieved from the ChromHL fit that corresponds to a significant energetic cost of nanodomain boundary formation. In line with this observation, nucleosome occupancy and distribution in ATRX-dependent HNDs were significantly different from other types of HNDs (Figs. 1D, 5B). Thus, thermodynamics of nucleosome packing is predicted to play a more important role in limiting the ATRX-dependent nanodomain size.

The DNA sequence dependence of HND initiation and extension raises the question, whether these domains are epigenetically regulated or represent mostly constitutive heterochromatin. Since ChromHL explicitly includes chromatin ligand binding, one straightforward mechanism for cell type-specific HND formation would be to epigenetically regulate the activity/concentration of the nucleation factors, CTCF or histone methylases that set the H3K9me2/3 mark. In addition, we show here how the change of the HP1 concentration and corresponding nucleosome occupancy induces the shrinking/merging of nanodomains, which could drive cell-type-specific differences (Fig. 6A, B Supplementary Fig. 15). Thus, by modulating the local concentrations of proteins that initiate (e.g., PAX3/9, ADNP), stop (e.g., CTCF) or promote the spreading of chromatin nanodomains (e.g., HP1), the cell can regulate the epigenetic states despite the DNA sequence contribution for defining nucleation sites and boundaries (Fig. 6C). In addition, ADNP and CTCF can compete for binding sites^51^, which could contribute to a modulation of ADNP domain size extension in dependence of the ADNP/CTCF binding activity ratio. CTCF (and other chromatin proteins) also bind in competition with nucleosomes that can adopt different positions as reflected in the NRL analysis conducted here. Accordingly, cell-type-specific binding of CTCF to certain sites is driven by a complex interplay of nucleosome binding, DNA (de)methylation and other factors^29,52^.

The mechanism of HND formation described here could also be relevant for the formation of larger heterochromatin domains at repetitive sequences. For example, pericentric heterochromatin in mouse cells comprises several Mb on a given chromosome with SUV39H being responsible for more than 50% of the H3K9me3 modification at this region^24^. As it consists predominantly of repetitive major satellite sequences, it is well conceivable that these very large H3K9me3 domains arise from the fusion of HNDs that form in a repetitive manner.

In summary, the ChromHL modelling approach developed here identified key parameters for the description of HNDs that are abundant in the genome. The analysis has been conducted for mouse ESCs but can be applied to other cell types and chromatin nanodomain types as well. For example, it is straightforward to include in ChromHL analysis HND types dependent on KRAB Zink finger proteins^53^. Thus, we anticipate that ChromHL will be valuable to distinguish sequence- and non-sequence-dependent contributions to establishing cell-type-specific chromatin state patterns and gene expression programs.

## Methods

### Cell lines and cell culture work

Wild type murine embryonic stem cells (ESCs) wt26 and *Atrx* knock out cell lines (KO1-40 and KO1-45) were described previously^32^. Cells were cultured on 0.2% v/v gelatine (in PBS) in high glucose DMEM (Gibco 31053-028) supplemented with 1 mM sodium pyruvate and 4 mM L-glutamine (PAA M11-006), 15% v/v FCS (Sigma F7524, lot: 091M3398), 1% v/v penicillin-streptomycin (PAN Biotech P06-07100), 100 µM β-mercaptoethanol (Sigma 63689), 1% v/v non-essential amino acids and 0.41% v/v LIF prepared from the supernatant from LIF-producing cells.

### ChIP-seq to map ATRX HNDs

ChIP-seq experiments were conducted essentially as described before^54^. To shear the chromatin, cells were digested with MNase for 15 min in a buffer containing 25 mM KCl, 4 mM MgCl_2_, 1 mM CaCl_2_, 50 mM Tris/HCl pH 7.4 and 1× protease inhibitor from Cell Signalling and sonicated with a Covaris S2 sonicator (parameters: 900 s, burst 200, cycle 20%, intensity 8) in sonication buffer (10 mM Tris pH 8.0, 200 mM NaCl, 1 mM EDTA, 0.5% N-lauroylsarcosine, 0.1% Na-deoxycholate). For pre-clearance, 4 µg normal rabbit IgG (R&D Systems, AB-105-C, lot: ER1212071) and ChIP-grade protein G magnetic beads (Cell Signalling 9006 S, 25 µl/sample) were used. A 1/20 fraction of the supernatant was used as input sample and the remaining material was split for three IP reactions with an anti-H3K9me3 antibody (Abcam, ab8898, lot: GR148830-2), an anti-histone H3 rabbit polyclonal antibody (Abcam, ab1791, lot: GR103864-1) and rabbit IgG (same as above). An amount of 4 µg antibody was added to each IP sample and incubated at 4 °C for 2 h. Then protein G magnetic beads were added and then the mixture was incubated at 4 °C overnight. After elution of the IP samples, cross-linking was reversed, RNase A and proteinase K were added, and DNA was precipitated. Experiments were conducted for two replicates of the wild-type cell line (wt26) and one replicate of each *Atrx* ko cell line (KO1-40 and KO1-45). Libraries for ChIP-seq were prepared with the NEBNext Ultra DNA Library Prep Kit for Illumina (New England Biolabs, NEB #E7370) according to the manufacturer’s instructions. In the size selection step, 150 bp fragments were selected that corresponded to 270 bp total size when including adaptor sequences. A total of 13 PCR amplification cycles were carried out and the library size and quality were checked by gel electrophoresis.

### Analysis of ATRX-dependent H3K9me3 ChIP-seq

Sequencing was performed on an Illumina HiSeq 2000 platform at the DKFZ Sequencing Core Facility. All samples were sequenced with 50-bp single-end reads and mapped to the mouse genome mm9 with Bowtie2^55^ allowing up to 2 mismatches (Supplementary Fig. 1 and Reporting Summary). Only uniquely mappable reads were retained. Thus, our analysis did not cover large repetitive regions but rather focused on the interspersed HND domains found outside the large blocks of constitutive H3K9me2/3 heterochromatin as present for example at the pericentric heterochromatin.

### Peak calling of H3K9me2/3 HNDs from experimental data

For a given HND type, differential H3K9me3 or H3K9me2 peaks were called with MACS2 (Zhang et al.^56^) of the WT and KO datasets of *Suv39h1/h2* ^*3*^ (GSE40086), *Glp*^*5*^ (GSE54412), and *ATRX* (determined in this study), respectively, against the common input and using the parameter broad-cutoff = 0.1. Peaks present in WT but not in the KO cells were retained as peaks that were dependent on a given factor. In the case of ADNP-associated HNDs a H3K9me3 dataset in *Adnp*^−/−^ cells was not reported^6^. Therefore, we defined ADNP-associated HNDs as the intersection of ADNP-bound ChIP-seq peaks with all H3K9me3 peaks in wild-type ESCs from these experiments (GSE97945) (*n* = 4673). Manipulations with BED files were performed using BEDTools^57^. For H3K9me3 in NPCs, we used datasets GSE61874^58^ and GSE57092^4^ with peak calling performed by MACS for Supplementary Fig. 15 as well as EPIC^59^ for Fig. 6B. We then intersected H3K9me3 peaks in ESCs and NPCs and retained in the analysis only those peaks which overlapped between these two conditions. The intersections between the four types of HNDs considered in this work are shown in Supplementary Fig. 19.

### Bioinformatical analysis of chromatin features

Our chromatin annotation used the 15 chromatin states assigned to ESC genomic regions previously^45^ using the package ChromHMM^60^. The nucleosome repeat length (NRL) was determined based on the previously published MNase-seq dataset^54^ (GSE40910) using NucTools^43^. The dyad-dyad differences were computed using the chemical mapping dataset^44^ (GSE82127). The Kolmogorov-Smirnov test *p*-values for the histograms of the dyad-dyad distance distributions were calculated using OriginPro software (OriginLab). In addition to H3K9me3 and H3K9me2 (datasets detailed above), the aggregate landscapes of nucleosome occupancy^54^ (GSE40910), binding of CTCF^61^ (GSE29184) and HP1^4^ (GSE57092), mappability, CpG methylation^62^ (GSE30206), and H3K4me1 and H3K27me3 modifications^61^ (GSE29184) were computed on regions of 40,000 bp centred on the HND centre using NucTools^43^. These were further smoothed using a 2000 bp Savitsky-Golay filter of order 2. Enrichments were computed with BEDTools as the ratio of the observed number of intersections between a given datasets and a given genomic feature to the number expected by chance for the same number of randomly shuffled regions. Mappability profiles around HNDs were calculated using the 36-nucleotide mappability track downloaded from the UCSC Genome Browser. This track was created by Thomas Derrien and Paolo Ribeca in Roderic Guigo’s lab at the Centre for Genomic Regulation (Barcelona) and shows how uniquely k-mer sequences align to a region of the genome.

### Identification of HND nucleation sites

In the case of SUV39H- and GLP-dependent heterochromatin we scanned the DNA sequences using RSAT^63^ with the PAX3 and PAX9 position weight matrix (PWM) obtained from TRANSFAC^64^ to obtain the locations of the motif within each peak. In the case of ATRX-dependent heterochromatin we obtained the list of specific L1 peaks (L1Md_F2, L1Md_T, L1Md_A and L1Md_F) using the RepeatMasker tool from the UCSC Genome Browser^65^. In the case of ADNP-associated HNDs we derived the PWM for sequence-specific ADNP binding using MEME^66^ based on 100-bp summits of 600 top ADNP-bound ChIP-seq peaks from Ostapcuk et al.^6^ (Supplementary Table 4). This PWM was then used for DNA sequence scanning with RSAT to determine ADNP motif locations inside H3K9me3 ChIP-seq peaks intersecting with ADNP-bound ChIP-seq peaks. Telomeric repeats were defined as single telomeric repeat motif reported^35^ using RSAT^63^. G-quadruplex repeats were defined as regex search on the sequences for each ATRX dependent peak with fastaRegexFinder by Dario Beraldi to identify the DNA sequence motif (G_3_N_1-7_)_3_G_3_ (https://github.com/dariober/bioinformatics-cafe/tree/master/fastaRegexFinder).

### Formulation of the ChromHL model

Our approach to include epigenetic processes coupled to protein binding in chromatin is inspired by classical Ising-type models developed in 1970s. These models allowed it to combine DNA-ligand binding and DNA-melting in the same mathematical notation^67^. This concept is implemented as the chromatin hierarchical lattice or ChromHL model via an extension of the *MatrixUnwrap* transfer matrix formalism for quasi-equilibrium binding of proteins to nucleosome-containing DNA developed in our previous work^39,68,69^. The detailed model is described in the Supplementary Information. It accounts for different scales from nucleotide- to nucleosome-resolution. This hierarchy is represented by defining the lattice separately at the level of DNA base-pairs as lattice units for sequence-specific TF binding and at the level of nucleosomes as lattice units for chromatin state transitions. The general transfer matrix formalism^18,39,68,69^ considers the genomic binding landscape as an 1D lattice. Each lattice unit can be in a pre-defined number of states that depend on a limited number of neighbouring lattice units. For example, only the next neighbour unit in the limiting case of contact cooperativity or up to *V* next neighbour units in the more general case of chromatin looping accounted for in *MatrixUnwrap*^39^. The method works by assigning statistical weights to each combination of allowed states of a lattice unite number *i* given the state of the next unit *i* + 1. The matrix that stores these weights is called the transfer matrix. For many biological scenarios encountered in DNA-protein binding it is straightforward to construct equivalent solutions using both Markov Models and Ising models, as well as their transfer matrix formulations^69^. The transfer matrix formalism is advantageous to describe systems with increasing complexity because additional biological features can be included by adding new states to a transfer matrix initially constructed for simpler systems. In the *MatrixUnwrap* model, the maximum number of states *N* of a lattice unit is defined by the following formula: *N* = $\mathop{\sum }\limits_{g=1}^{f}({m}_{g}+{V}_{g})+2+\,\max ({V}_{g})+1$.

In this equation *f* is the number of protein types, *m*_g_ is the size if a protein complexes of type *g* and *V*_g_ is the maximum length of protein-protein interactions in which protein complex of type *g* can engage. In ChromHL, we increased the complexity by introducing chromatin states (*e*) of the lattice in addition to protein-binding states. In our calculations reported here, a lattice unit can belong to a nucleosome in a methylated or unmethylated state or can be in a nucleosome-free “insulator” state. Thus, the number of elementary states in *ChromHL* increases threefold in comparison with *N* protein-binding states in *MatrixUnwrap*. The statistical weight for each state of the elementary unit of the lattice is defined by multiplying the statistical weights of the constituent protein-bound states and the chromatin state (Supplementary Information).

### Calculations with the ChromHL model

ChromHL calculations are performed sequentially, first at the single-nucleotide resolution level and next at the single-nucleosome resolution level (Supplementary Fig. 18). The calculation at the single-nucleotide resolution level takes as input the DNA sequence and protein concentrations, weight matrices setting protein-DNA affinities and cooperativity constants for protein-protein binding. As output of this calculation, ChromHL generates maps of protein occupancies along the DNA, referred to as binding maps. These binding maps are then smoothed to coarse-grain the model to single-nucleosome resolution. An HND-initiation threshold is then applied to these binding maps. For models dependent on the binding of PAX3/9 as an initiation factor, if the binding affinity over the nucleosome is greater than a threshold (Supplementary Table 3), the element of the nucleosome-resolution lattice is assigned as HND-initiating. The three chromatin states (Fig. 1) are the methylated (*e* = 1) or unmethylated nucleosome (*e* = 2) and the absence of a nucleosome due to CTCF binding (*e* = 3). Outside of any CTCF binding or heterochromatin initiation site, we set *s*(*i*, 1) = *s*; *s*(*i*, 2) = 1; s(*i*, 3) = 0. The initiation of heterochromatin was specified in the model by setting *s*(*i*, 1) = 1, *s*(*i*, 2) = 0, *s*(*i*, 3) = 0, for the lattice unit *i* where initiation occurs. For the description of the artificial HP1 recruitment experiment the initiation site was at the centre point of the lattice. The weights of nucleosome-nucleosome contacts, σ(e_1_, e_2_) = σ_e1e2_, were simplified to setting the most important boundary parameter σ_12_ = σ_21_ = σ and all other contact weights equal to 1_._ Likewise, cooperativity parameters were set to 1, except for the contact HP1-HP1 cooperativity (Supplementary Fig. 12). The latter parameter was fitted in the case of the Hathaway et al experiments, or fixed in the case of in vivo heterochromatin formation to *w*(1, 1, 0) = 100 or *w*(1, 1, 0) = 1, as explained in Supplementary Fig. 4 and Supplementary Table 3. For TF-based heterochromatin initiation models the TRAP affinity score^70^ was computed using the corresponding PWM based on the DNA sequence. For PAX3/PAX9 binding, we used PAX3 PWM (JASPAR matrix MA0780.1) and PAX9 PWM (JASPAR matrix MA0781.1). The affinities calculated from PAX3 binding and PAX9 binding were combined additively. This affinity was then geometrically averaged over a 501-bp window and subjected to a threshold for including a lattice unit as a nucleation site if the averaged affinity for a given lattice unit was above the threshold (Supplementary Figs. 16, 17). For the ATRX-dependent heterochromatin, L1 sub-repeats L1Md_F2, L1Md_T, L1Md_A and L1Md_F (of approximately 200–300 bp) that were found to be enriched in the ATRX-dependent H3K9me3 peaks were downloaded from the RepeatMasker track on the UCSC Genome Browser (Supplementary Table 1). A lattice unit containing the centre of a repeat was considered an initiation site. The length of the lattice in units was calculated by placing a lattice unit exactly in the centre of the region and adding units either side of this position symmetrically until all the sequence was covered. The fit of the parameters was optimised by comparing the predicted and experimental distributions of HND sizes and minimising the distance between the two estimated distributions. A grid search in the parameter space was performed for this, as demonstrated in Supplementary Figs. 7–9.

### ChromHL computational performance

ChromHL calculations were conducted for 40 kb regions centred around a peak (20 kb on each side). For each 40 kb region, the calculation takes ~4 s on a Sun Grid Engine High Performance Cluster. Due to the independence of each region, the overall calculation can be conducted in parallel and subsequently aggregated. For example, the 36,764 Suv39h-dependent regions were split into 368 separate tasks containing up to 100 regions each (367 × 100 regions and 1 × 64 region), which were submitted as a 368-task batch job on a Sun Grid Engine High Performance Cluster running CentOS with ~1000 Xeon processors. This calculation took ~6 h to process from submission to aggregation.

### Receiver-operator curves (ROC) calculation

For SUV39H-dependent HNDs, we computed receiver-operator (ROC) curves using smoothed affinity scores on the peaks and matched non-peaks for different smoothing windows sizes (Supplementary Figs. 16B, D). The maximum area under the ROC curve (AUC) was calculated for 1001-bp windows. The AUC for this window size was not significantly different to that for 501 bp (Supplementary Figs. 16C, 16E). Thus, we used a 501 bp geometric mean centred smoothing window for determining heterochromatin initiation sites for these sets of peaks, assuming PAX3/9 binding is the initiation factor. For H3K9me3-dependent HNDS, the 36,764 H3K9me3 peaks in the SUV39H-dependent experiment were used as true positives. As true negatives, a set of 36,764 non-peaks was generated by shuffling the original peak locations with BedTools to obtain a matched dataset with the same interval sizes. For the combined set of intervals, we calculated base-pair-level affinities for the PAX3/9 transcription factors with the TRAP algorithm^70^. This value was smoothed by calculating the geometric mean using a centred window of different sizes (51, 101, 251, 501 bp). A true peak was scored as [1, maximum smoothed score] while a true negative result was scored as [0, maximum smoothed score]. The ROC curves were calculated for these pairs using the Origin Pro software (originlab.com).

### Reporting summary

Further information on research design is available in the Nature Research Reporting Summary linked to this article.

## Supplementary information

Supplementary Information

Reporting summary

## Data Availability

The ChIP-seq datasets from the ATRX knockout experiments generated in this study have been deposited in the GEO database under accession code GSE158744. The previously published datasets used in this study are available in the GEO database under accession codes GSE40086, GSE54412, GSE97945, GSE61874, GSE57092, GSE40910, GSE82127, GSE29184, GSE57092 and GSE30206 as detailed in Methods. Source data are provided with this paper.

## Code availability

The ChromHL software and associated codes are available at https://github.com/TeifLab/ChromHL^71^.
