# Supplementary material for: DNA sequence-dependent formation of heterochromatin nanodomains
Source: Nat Commun. 2022 Apr 6;13:1861. doi: 10.1038/s41467-022-29360-y (PMC8986797; doi:10.1038/s41467-022-29360-y)
Supplement: Supplementary file 1 — Supplementary Information [file 41467_2022_29360_MOESM1_ESM.pdf]

Supplementary Information

DNA sequence-dependent formation of heterochromatin nanodomains

Graeme J. Thorn[1,2], Christopher T. Clarkson[1,3], Anne Rademacher[4], Hulkar Mamayusupova[1], Gunnar Schotta[5], Karsten Rippe[4,*], Vladimir B. Teif[1,*]

[1]School of Life Sciences, University of Essex, Wivenhoe Park, Colchester, CO4 3SQ, UK.

[2]Current address: Barts Cancer Institute, Queen Mary University of London, Charterhouse Square, London, EC1M 6BQ, UK.

[3]Current address: University College London, Gower St, Bloomsbury, London WC1E 6BT, UK.

[4]Division of Chromatin Networks, German Cancer Research Center (DKFZ) & Bioquant, Heidelberg, 69120, Germany.

[5]Ludwig-Maximilians-Universität München, Geschwister-Scholl-Platz 1, 80539 München, Germany

*Correspondence should be addressed to Vladimir B. Teif (vteif@essex.ac.uk) or Karsten Rippe (karsten.rippe@dkfz.de).

Table of contents

Appendix A. Theoretical foundation of the ChromHL model Page 3

Appendix B. Algorithm for constructing the ChromHL transfer matrix Page 6

Appendix C. Supplementary figures and tables Page 12

Supplemental References Page 40

Appendix A. Theoretical foundation of the ChromHL framework

The Chromatin Hierarchical Lattice (ChromHL) framework is an extension of the transfer matrix models for combinatorial binding of proteins to DNA at thermodynamic equilibrium developed in our previous publications [1-6]. This formalism is motivated by classical Ising-type models [7] as was traditionally employed in DNA-binding and DNA-melting studies [8,9], considering chromatin as a quasi-1D lattice of units. Here we have extended the general transfer matrix formalism [1-5] to account for different scales from nucleotide- to nucleosome- to chromatin domain-resolution that are described in a hierarchical manner. We define the lattice separately at the level of DNA base-pairs as lattice units when sequence-specific TF binding is considered, and at the higher level of nucleosomes as lattice units when chromatin state transitions are considered.

At the nucleotide-resolution level, we take as input the DNA sequence, protein-DNA binding preferences in the form of weight matrices and protein concentrations. In addition, thermodynamic parameters defining their interaction with DNA and with each other, as well as geometric parameters defining the number of DNA base pairs covered upon binding go into the model. Our previous publications [1-4] established the way to take into account cooperative combinatorial binding of multiple types of proteins to nucleosomal DNA. As detailed below, short-range and long-range interactions and the possibility of incomplete binding (e.g. in the case of partial unwrapping of DNA from the nucleosome or other protein complexes) can be included [10]. As output, at this level of hierarchy we predict binding maps for each protein species with single-nucleotide resolution. Some of the binding proteins act as nucleation points for chromatin nanodomains, which provides the connection to the next level of hierarchy.

At the nucleosome-resolution level we assume that the interaction between the neighboring nucleosomes depends on the characteristic nucleosome repeat lengths (NRLs), nucleosome decoration with different histone modifications as well as other structural effects which are all cast in the phenomenological definition of the “chromatin state” (Figure 1). A nucleosome can belong to any chromatin state, characterized by a certain self-energy depending on the given state, a nucleosome-nucleosome interaction energy depending of the states of the two nucleosomes, and a protein binding energy depending on the protein type and the nucleosome state. Proteins bound to neighboring nucleosomes can interact with each other. Previously we have constructed a mechanistic model of heterochromatin protein 1 (HP1) binding to the nucleosome array [5] based on *in vitro* binding experiments [11]. Here we add to the HP1 model additional players such as CTCF, PAX3, PAX9 and ADNP capable to initiate heterochromatin nanodomains. We parameterize this model based on the published experimental datasets for mouse embryonic stem cells (ESCs) with respect to targeted recruitment of HP1 [12], Suv39h1/h2-dependent H3K9me3-marked heterochromatin [13], GLP-dependent H3K9me3-marked heterochromatin [14], ADNP-dependent H3K9me3 heterochromatin [15] as well as a new data set introduced here on ATRX-dependent H3K9me3 heterochromatin.

The mathematical solution of the ChromHL model is based on the general transfer matrix formalism [1,2,4-6]. In this method each elementary lattice unit can be in a number of states and can be affected only by a limited number of neighboring lattice units (e.g. just the next neighbor unit in the limiting case of contact cooperativity, or up to V next neighbor units in the more general case accounted for in our previous publications) [1,4,6,16,17]. All states need to be enumerated and the corresponding statistical weights need to be assigned for all combinations of allowed states of a lattice unite number i given the state of the next unit i+1. The matrix that stores these weights is called the transfer matrix. In our previous publication

a transfer matrix called *MatrixUnwrap* has been already constructed at the single-base pair level for a model that takes into account cooperative competitive TF binding to the DNA inside and outside the nucleosome and longer than nearest-neighbor interactions [4,6]. Here we increased the complexity of the model by introducing additional arbitrary states of the lattice (e.g., belonging to a nucleosome in a “heterochromatin” or “euchromatin” state, or belonging to a nucleosome-free “insulator region”).

To illustrate the general matrix solution of this problem, consider the simple case of a non-sequence-specific protein binding to DNA that is characterized by contact cooperativity. In this case, each lattice unit can be in two states: bound or unbound, and the corresponding transfer matrix *A* for is 2x2:

$$A = \begin{pmatrix} wKc & Kc \\ 1 & 1 \end{pmatrix},$$

where each row corresponds to the state of a given lattice unit *i*, and each column to the state of the next lattice unit *i*+1, *K* is the binding constant, *c* is the free protein concentration, *w* is the contact cooperativity between DNA-bound proteins. In this case we assigned 1 as the weight of a lattice unit that is not bound by protein. The weight of a lattice unit bound by the protein is *Kc*. The weight of a lattice unit bound by a protein interacting with the next lattice unit that is also bound by a protein is given as a product $w \cdot K \cdot c$, where *w* is the contact cooperativity constant.

Let us also consider another simple case of a lattice where each lattice unit, say, a nucleosome, can be in two states: say, heterochromatin, and euchromatin. Let us characterize each of these states by a weight s_1 and s_2 correspondingly. Let the interaction between two nucleosomes is characterized by a weight σ that depends on the state of each of these two nucleosomes. In this case the transfer matrix *B* for such lattice will have a simple form:

$$B = \begin{pmatrix} s_1\sigma_{11} & s_1\sigma_{12} \\ s_2\sigma_{21} & s_2\sigma_{22} \end{pmatrix}$$

When combining the two effects (protein binding reflected in matrix *A* and nucleosome s_1/s_2 transition reflected in matrix *B*), each lattice unit will have four possible states (protein-bound s_1; unbound s_1, protein-bound s_2, unbound s_2). The corresponding transfer matrix *C* will be 4x4 as summarized below:

$$C = \begin{pmatrix} w_{11}K_1c_1s_1\sigma_{11} & K_1c_1s_1\sigma_{11} & w_{12}K_1c_1s_1\sigma_{12} & K_1c_1s_1\sigma_{12} \\ s_1\sigma_{11} & s_1\sigma_{11} & s_1\sigma_{12} & s_1\sigma_{12} \\ w_{12}K_2c_2s_2\sigma_{21} & K_2c_2s_2\sigma_{21} & w_{22}K_2c_2s_2\sigma_{22} & K_2c_2s_2\sigma_{22} \\ s_2\sigma_{21} & s_2\sigma_{21} & s_2\sigma_{22} & s_2\sigma_{22} \end{pmatrix}$$

In principle, we can distinguish free protein concentration *c* inside chromatin regions in state 1 and 2, denoted correspondingly c_1 and c_2. Different chromatin states can have different free concentrations of our protein of interest if it is locally confined in a nuclear subcompartment [18].A system characterised by the simple transfer matrix C above can be solved even analytically, deriving exact expressions for the average size of the heterochromatin nanodomain, average number of nanodomain boundaries, etc. However, in reality protein binding is sequence specific and depends on the chromatin states. Thus, the parameters *s* and *K* are the functions of the location of the lattice unit along the genomic coordinate (*n*), which precludes analytical solutions. To construct a DNA sequence-informed transfer matrix (and also taking into account more complicated scenarios of cooperative

combinatorial binding), we base the ChromHL methodology on a more advanced transfer matrix. A transfer matrix called *MatrixUnwrap* developed previously [4] is used instead of matrix *A* shown above. The *MatrixUnwrap* model is defined for protein-DNA binding that takes into account binding of multiple types of proteins g (g = 1…f) at concentrations $c(g)$, which bind lattice units in different states e_n with binding constants $K(n, g, e_n)$, and interact with each other with distance-dependent potential $w(g_1, g_2, l)$ ($l < V_g$). Furthermore, this transfer matrix accounts for the partial unwrapping of DNA from the nucleosome, and that DNA-bound proteins can have incomplete-binding states when not all protein-DNA interactions are formed. The total number of states in the *MatrixUnwrap* model is given by the following expression:

$$\sum_{g=1}^{f}\left(m_g + V_g\right) + 2 + \max(V_g) + 1$$

In addition to the MatrixUnwrap states, we introduce e_{max} additional chromatin states e_n for each lattice unit (e_n=[1,e_{max}]). (e.g. a nucleosome can be in heterochromatin state or euchromatin state). Correspondingly, each individual nucleosome is characterized by the weight $s(e_n) = \exp(\Delta G(e_n)/RT)$, where $\Delta G(e_n)$ is the energy of the nucleosome in state e_n, R is the universal gas constant, T is the temperature. Similarly, the contact between two neighboring nucleosomes in states e_1 and e_2 is assigned a weight $\sigma(e_{n,} e_2) = \exp(\Delta G(e_{1,}e_2)/RT)$, where $\Delta G(e_{1,}e_2)$ is the energy of nucleosome-nucleosome interaction. This results in the increase of the total number of states of the model as follows:

$$e_{\max} \cdot \left[\sum_{g=1}^{f}\left(m_g + V_g\right) + 2 + \max(V_g) + 1\right]$$

A detailed description of the assignment of all transfer matrix states is detailed below in Appendix B.

Once sequence-dependent transfer matrices Q_n are constructed for each lattice unit, we can calculate the partition function Z of the lattice of length N by multiplying all transfer matrices Q_n sequentially:

$$Z = \left(1\ 1 \ldots 1\right) \times \prod_{n=1}^{N} Q_n \times \begin{pmatrix} 1 \\ 1 \\ \ldots \\ 1 \end{pmatrix}$$

The probability that a given lattice unit n is in state x can be calculated as

$$P_n(X) = \frac{s_{xn}}{NZ} \times \frac{\partial Z}{\partial s_{xn}},$$

where s_{xn} is the statistical weight for a state x for a given lattice unit. In particular, we can calculate the binding maps for any protein of interest, as well as the maps of "epigenetic" states for any DNA sequence and concentrations of regulatory proteins such as HP1.

Appendix B. Algorithm for constructing the ChromHL transfer matrix

The parameters in the transfer matrix are defined as follows:

n	Genomic coordinate of the lattice unit
g	Type of protein (e.g. types of transcription factors)
f	Maximum number of types of proteins that bind the DNA lattice
$c_0(g)$	Concentration of free proteins of type g
e	Type of the chromatin state for a given lattice unit (e.g. heterochromatin or euchromatin)
e_{max}	Maximum number of chromatin states considered in the model
$\sigma(e_i, e_j)$	Statistical weight of forming a boundary between different chromatin states e_i and e_j.<br><br>$\sigma(e_i, e_j) = e^{-\Delta F(e_i, e_j)/RT}$, where *R* is the universal gas constant, *T* is the temperature, $\Delta F(e_i, e_j)$ is the energy difference between interaction of nucleosomes in different states e_i, and e_j versus same-state interactions. In the calculations performed in the current manuscript, same-state interactions are set as $\sigma(e_i, e_i) = 1$.
$s(e_i, e_j)$	Statistical weight of switching chromatin state from e_i to e_j. $s(e_i, e_j) = e^{\Delta G(e_i, e_j)/RT}$, where $\Delta G(e_i, e_j)$ is the energy difference between chromatin states e_i, and e_j of the lattice unit.
m_g	Maximum number of lattice units covered by protein of type *g* upon binding
h	Number of DNA lattice units unwrapped from the bound protein, $h < m(g)$
$K(n, g, p, e_i)$	Microscopic binding constant for forming a contact between unit *p* of protein g and DNA lattice unit *n* in chromatin state e_i. In the case of homogeneous binding the microscopic binding constant $K(n, g, p, e_i)$ is related to the macroscopic binding constant of a protein as a whole, $K(n, g, e_i)$, as follows: $K(n, g, p, e_i) = \sqrt[m_g]{K(n, g, e_i)}$
V_g	Maximum interaction distance between two proteins bound to the lattice considered in the model (the number of lattice units between the proteins)
$w(l, g_1, g_2, e_i)$	Statistical weight for interaction between proteins g_1 and g_2 separated by l lattice units, $l < max(V_g)$, in chromatin state e_i. $g_1, g_2 = 0$ is reserved for the absence of bound protein.
$U(h, g)$	Statistical weight for the chance of unwrapping *h* DNA lattice units from bound protein g. In the calculations performed in the current manuscript partial unwrapping was prohibited.

The full transfer matrix is square block structured:

$$Q_n = \begin{pmatrix} Q_n^{1,1} & \cdots & Q_n^{1,e_{max}} \\ \vdots & \ddots & \vdots \\ Q_n^{e_{max},1} & \cdots & Q_n^{e_{max},e_{max}} \end{pmatrix},$$

with $e_{max} \times e_{max}$ blocks of individual inner transfer matrices $Q_n^{e_i,e_j}$ representing transitions between chromatin state e_i and $e_j$at lattice unit n.

Within each inner block $Q_n^{e_i,e_j}$, the states are enumerated as follows:

State i for lattice unit n	**State description**	
	type of complex	position of unit
1	1	1
$\cdots$		$\cdots$
m_1		m_1
$\cdots$	$\cdots$	$\cdots$
$\sum_1^{g-1} m_k + 1$	g	1
$\cdots$		$\cdots$
$m_1 + \cdots + m_g$		m_g
$\cdots$	$\cdots$	$\cdots$
$m_1 + \cdots + m_{f-1} + 1$	f	1
$\cdots$		$\cdots$
$m_1 + \cdots + m_f$		m_f
$\sum_{g=1}^{f} m_g + 1$	left free DNA end	
$\sum_{g=1}^{f} m_g + 2$	right free DNA end	
$\cdots$	$\cdots$	
$\sum_{g=1}^{f} m_g + 2 + \sum_{g=1}^{g_2-1} V_g + l$	g_1-l-g_2 gap (<br>l free units before next g_2 protein), $l \le V_{g2}$	
$\cdots$	$\cdots$	
$\sum_{g=1}^{f} (m_g + V_g) + 2 + l$	g_1-l-g_2 (l free units before next g_2 protein), $l > V_{g2}$	
$\cdots$	$\cdots$	
$\sum_{g=1}^{f} (m_g + V_g) + 2 + max(V_g) + 1$	free unit out of protein-protein interactions, not at the DNA ends	

As there are $e_{max} \times e_{max}$ blocks in the full transfer matrix, the total number of states in Q_n is $e_{max} \times \left(\sum_{g=1}^{f}(m_g + V_g) + 2 + max(V_g) + 1\right)$.

For each pair of chromatin states e_1 and e_2, ($e_1, e_2 = 1 \ldots e$) the inner block transfer matrix $Q_n^{e_1,e_2}$ is built up in the following way:

1) 1st unit of g-type protein followed by the 2nd unit:

$$1 \le g \le f,\ i = \sum_{k=1}^{g-1} m_k + 1,\ j = i + 1:$$

$$\begin{cases} n = 1: & Q_n^{e_1 e_2}(i,j) = s(n,e_1) \times \sigma(e_1,e_2) \times K(n,g,1,e_1) \times c_0(g) \\ 1 < n \le N - (m_g - 1): & Q_n^{e_1 e_2}(i,j) = s(n,e_1) \times \sigma(e_1,e_2) \times K(n,g,1,e_1) \end{cases} \quad (1)$$

2) Unit $h + 1$ of g-type protein following by unit $h + 2$ (if $m > 2$):

$$1 \le g \le f,\ i = \sum_{k=1}^{g-1} m_k + 1,\ j = i + 1,\ 1 < h \le m_g - 2,\ h < n \le N - (m - h - 1):$$

$$Q_n^{e_1 e_2} = s(n,e_1) \times K(n,g,h+1,e_1). \quad (2)$$

3) Unit ($m_g - h$) of g-type protein followed by a right free DNA end:

a) if $m_g = 1$:

$$1 \le g \le f,\ i = \sum_{k=1}^{g} m_k - h,\ 0 \le h < m_g,\ j = \sum_{g=1}^{f} m_g + 2,\ m_g - h \le n \le N - h\ :$$

$$\begin{cases} n = 1: & Q_n^{e_1 e_2}(i,j) = s(n,e_1) \times \sigma(e_1,e_2) \times K(n,g,m_g - h,e_1) \times w(0,g_1,0,e_1) \times c_0(g) \\ n > 1: & Q_n^{e_1 e_2}(i,j) = s(n,e_1) \times \sigma(e_1,e_2) \times K(n,g,m_g - h,e_1) \times w(0,g_1,0,e_1) \end{cases} \quad (3a)$$

b) if $m_g > 1$:

$$1 \le g \le f,\ i = \sum_{k=1}^{g} m_k - h,\ 0 \le h < m_g,\ j = \sum_{g=1}^{f} m_g + 2,\ m_g - h \le n \le N - h\ :$$

$$\begin{cases} n = 1: & Q_n^{e_1 e_2}(i,j) = s(n,e_1) \times \sigma(e_1,e_2) \times K(n,g,m_g - h,e_1) \times U(h,g) \times c_0(g) \\ n > 1: & Q_n^{e_1 e_2}(i,j) = s(n,e_1) \times \sigma(e_1,e_2) \times K(n,g,m_g - h,e_1) \times U(h,g) \end{cases} \quad (3b)$$

4) Unit ($m_{g1} - h_1$) of g_1-type protein followed by unit 1 of g_2-protein (no gap):

a) if $m_{g1} = 1$:

$$1 \le g_1 \le f,\ i = \sum_{k=1}^{g_1} m_k - h_1,\ 0 \le h_1 < m_{g1},\ 1 \le g_2 \le f,\ j = \sum_{k=1}^{g_2 - 1} m_k + 1,$$

$$m_{g1} - h_1 \le n \le N - m_{g2}:$$

$$\begin{cases} n = 1: & Q_n^{e_1e_2}(i,j) = s(n,e_1) \times \sigma(e_1,e_2) \times K(n,g_1,m_{g1}-h,e_1) \times w(0,g_1,g_2,e_1) \times c_0(g_1) \times c_0(g_2) \\ n > 1: & Q_n^{e_1e_2}(i,j) = s(n,e_1) \times \sigma(e_1,e_2) \times K(n,g_1,m_{g1}-h,e_1) \times w(0,g_1,g_2,e_1) \times c_0(g_2) \end{cases} \quad (4a)$$

b) if $m_{g1} > 1$:

$$1 \le g_1 \le f,\ i = \sum_{k=1}^{g_1} m_k - h_1,\ 0 \le h_1 < m_{g1},\ 1 \le g_2 \le f,\ j = \sum_{k=1}^{g_2-1} m_k + 1,$$

$$m_{g1} - h_1 \le n \le N - m_{g2}: \quad (4b)$$

$$\begin{cases} n = 1: & Q_n^{e_1e_2}(i,j) = s(n,e_1) \times \sigma(e_1,e_2) \times K(n,g_1,m_{g1}-h,e_1) \times w(0,g_1,g_2,e_1) \times U(h,g) \times c_0(g_1) \times c_0(g_2) \\ n > 1: & Q_n^{e_1e_2}(i,j) = s(n,e_1) \times \sigma(e_1,e_2) \times K(n,g_1,m_{g1}-h,e_1) \times w(0,g_1,g_2,e_1) \times U(h,g) \times c_0(g_2) \end{cases}$$

5) Last unit of g_1-type protein followed by unit $h_2 + 1$ of g_2-protein (no gap):

a) if $m_{g1} = 1$:

$$1 \le g_1 \le f,\ i = \sum_{k=1}^{g_1} m_k,\ 1 \le g_2 \le f,\ j = \sum_{k=1}^{g_2-1} m_k + 1 + h_2,\ 1 \le h_2 < m_{g2}, m_{g1} \le n \le N - m_{g2} - h_2:$$

$$\begin{cases} n = 1: & Q_n^{e_1e_2}(i,j) = s(n,e_1) \times \sigma(e_1,e_2) \times K(n,g_1,m_{g1},e_1) \times w(0,g_1,g_2,e_1) \times c_0(g_1) \times c_0(g_2) \\ n > 1: & Q_n^{e_1e_2}(i,j) = s(n,e_1) \times \sigma(e_1,e_2) \times K(n,g_1,m_{g1},e_1) \times w(0,g_1,g_2,e_1) \times c_0(g_2) \end{cases} \quad (5a)$$

b) if $m_{g1} > 1$:

$$1 \le g_1 \le f,\ i = \sum_{k=1}^{g_1} m_k,\ 1 \le g_2 \le f,\ j = \sum_{k=1}^{g_2-1} m_k + 1 + h_2,\ 1 \le h_2 < m_{g2}, m_{g1} \le n \le N - m_{g2} - h_2:$$

$$Q_n^{e_1e_2}(i,j) = s(n,e_1) \times \sigma(e_1,e_2) \times K(n,g_1,m_{g1},e_1) \times w(0,g_1,g_2,e_1) \times U(h_2,g_2) \times c_0(g_2) \quad (5b)$$

6) Left free DNA end continues:

$$i = j = \sum_{g=1}^{f} m_g + 1:\ Q_n^{e_1e_2}(i,j) = s(n,e_1) \times \sigma(e_1,e_2) \times w(0,0,0,e_1) \quad (6)$$

7) Right free DNA end continues:

$$i = j = \sum_{g=1}^{f} m_g + 2, n > 1:\ Q_n^{e_1e_2}(i,j) = s(n,e_1) \times \sigma(e_1,e_2) \times w(0,0,0,e_1) \quad (7)$$

8) Left free end followed by unit *h*+1 of *g*-type protein:

$$1 \le g \le f,\ i = \sum_{g=1}^{f} m_g + 1,\ j = \sum_{k=1}^{g-1} m_k + 1 + h,\ 0 \le h < m_g, n \le N - (m_g - h):$$

$$Q_n^{e_1e_2}(i,j) = s(n,e_1) \times \sigma(e_1,e_2) \times w(0,0,g,e_1) \times U(h,g) \times c_0(g) \quad (8)$$

9) Unit ($m_g - h$) of g-type protein followed by a non-interacting gap longer than V_g:

a) if $m_g = 1$:

$$1 \le g \le f,\ i = \sum_{k=1}^{g} m_k - 1 - h,\ 0 \le h < m_g,\ j = \sum_{g=1}^{f}(m_g + V_g) + 2, m_g - h \le n \le N:$$

$$\begin{cases} n = 1: & Q_n^{e_1e_2}(i,j) = s(n,e_1) \times \sigma(e_1,e_2) \times K(n,g,m_g,e_1) \times w(V_g + 1, g, 0, e_1) \times U(h,g) \times c_0(g) \\ n > 1: & Q_n^{e_1e_2}(i,j) = s(n,e_1) \times \sigma(e_1,e_2) \times K(n,g,m_g,e_1) \times w(V_g + 1, g, 0, e_1) \times U(h,g) \end{cases} \quad (9a)$$

b) if $m_g > 1$:

$$1 \le g \le f,\ i = \sum_{k=1}^{g} m_k - 1 - h,\ 0 \le h < m_g,\ j = \sum_{g=1}^{f}(m_g + V_g) + 2, m_g - h \le n \le N:$$

$$\begin{cases} n = 1: & Q_n^{e_1e_2}(i,j) = s(n,e_1) \times \sigma(e_1,e_2) \times K(n,g,m_g,e_1) \times w(V_g + 1, g, 0, e_1) \times c_0(g) \\ n > 1: & Q_n^{e_1e_2}(i,j) = s(n,e_1) \times \sigma(e_1,e_2) \times K(n,g,m_g,e_1) \times w(V_g + 1, g, 0, e_1) \end{cases} \quad (9b)$$

10) Large non-interacting gap continues, units before $max(V_g)$:

$$i = \sum_{g=1}^{f}(m_g + V_g) + 2, 1 \le k \le \max(V_g), j = i + k:\ \ Q_n^{e_1e_2}(i,j) = s(n,e_1) \times \sigma(e_1,e_2) \quad (10)$$

11) Large non-interacting gap continues, units after $max(V_g)$:

$$i = j = \sum_{g=1}^{f}(m_g + V_g) + 2 + \max(V_g) + 1, 1 < n < N:\ \ Q_n^{e_1e_2}(i,j) = s(n,e_1) \times \sigma(e_1,e_2)$$

(11)

12) Large non-interacting gap followed by unit $h + 1$ of g-type protein:

$$i = \sum_{g=1}^{f}(m_g + V_g) + 2 + \max(V_g) + 1, j = \sum_{k=1}^{g-1} m_k + 1 + h, 0 \le h < m_g, 1 < n < N - (m_g - h):$$

$$Q_n^{e_1e_2}(i,j) = s(n,e_1) \times \sigma(e_1,e_2) \times w(\max(V_g) + 1, 0, g, e_1) \times Unwrap(h,g) \times c_0(g) \quad (12)$$

13) Unit ($m_{g1} - h$) of g_1-type protein followed by l-gap followed by g_2-protein:

a) if $m_{g1} = 1$:

$$i = \sum_{k=1}^{g_1} m_k - h, 0 \le h < m_{g1},\ j = \sum_{g=1}^{f} m_g + 2 + \sum_{g=1}^{g_2-1} V_g + l,\ 1 \le l \le V_{g2} \text{ for } V_{g2} > 0,\ m_{g1} \le n \le N:$$

$$\begin{cases} n = 1: & Q_n^{e_1 e_2}(i,j) = s(n,e_1) \times \sigma(e_1,e_2) \times K(n,g,m_{g1},e_1) \times w(l,g_1,g_2,e_1) \times c_0(g) \\ n > 1: & Q_n^{e_1 e_2}(i,j) = s(n,e_1) \times \sigma(e_1,e_2) \times K(n,g,m_{g1},e_1) \times w(l,g_1,g_2,e_1) \times c_0(g) \end{cases} \quad (13a)$$

b) if $m_{g1} > 1$:

$$i = \sum_{k=1}^{g_1} m_k - h, 0 \le h < m_{g1}, \; j = \sum_{g=1}^{f} m_g + 2 + \sum_{g=1}^{g_2-1} V_g + l, \; 1 \le l \le V_{g2} \text{ for } V_{g2} > 0, \; m_{g1} \le n \le N:$$

$$\begin{cases} n = 1: & Q_n^{e_1 e_2}(i,j) = s(n,e_1) \times \sigma(e_1,e_2) \times K(n,g,m_{g1} - h,e_1) \times w(V_g + 1,g,0,e_1) \times U(h,g) \times c_0(g) \\ n > 1: & Q_n^{e_1 e_2}(i,j) = s(n,e_1) \times \sigma(e_1,e_2) \times K(n,g,m_{g1} - h,e_1) \times w(V_g + 1,g,0,e_1) \times U(h,g) \end{cases}$$

(13b)

14) g_1-l-g_2 gap continues (l free units before g_2-type protein):

$$2 \le l \le V_{g2} \text{ for } V_{g2} > 0, \; i = \sum_{g=1}^{f} m_g + 2 + \sum_{g=1}^{g_2-1} V_g + l, j = i - 1, 1 \le n \le N: \; Q_n^{e_1 e_2}(i,j) = s(n,e_1) \times \sigma(e_1,e_2)$$

(14)

15) g_1-l-g_2 gap followed by unit $h + 1$ of g_2 protein:

$$i = \sum_{g=1}^{f} m_g + 2 + \sum_{g=1}^{g_2-1} V_g, j = \sum_{k=1}^{g_2-1} m_k + 1 + h, \; 0 \le h \le m_{g2}, 1 < n < N - m_{g2} V_g \; :$$

$$Q_n^{e_1 e_2}(i,j) = s(n,e_1) \times \sigma(e_1,e_2) \times Unwrap(h,g_2) \times c_0(g_2) \quad (15)$$

Following standard mathematical notations, in all equations above we assume that the sum equals 0 in the case if the upper summation limit is less than the lower limit.

The partition function Z and its derivatives are calculated recursively:

$$Z = A_N \times \begin{pmatrix} 1 \\ 1 \\ \dots \\ 1 \end{pmatrix}, \quad A_i = A_{i-1} \times Q_n \quad (16)$$

$$\frac{\partial Z}{\partial K(n,g)} = \frac{\partial A_N}{\partial K(n,g)} \times \begin{pmatrix} 1 \\ 1 \\ \dots \\ 1 \end{pmatrix}, \quad \frac{\partial A_n}{\partial K(n,g)} = \frac{\partial A_{n-1}}{\partial K(n,g)} \times Q_n + A_{n-1} \times \frac{\partial Q_n}{\partial K(n,g)},$$

$$\frac{\partial Z}{\partial s(n,e)} = \frac{\partial A_N}{\partial s(n,e)} \times \begin{pmatrix} 1 \\ 1 \\ \dots \\ 1 \end{pmatrix}, \quad \frac{\partial A_n}{\partial s(n,e)} = \frac{\partial A_{n-1}}{\partial s(n,e)} \times Q_n + A_{n-1} \times \frac{\partial Q_n}{\partial s(n,e)},$$

$$A_0 = (1 \quad 1 \quad \dots \quad 1) \quad (17)$$

Since the elements of the transfer matrix only depend linearly on $K(n,g)$ and $s(n,e)$, the probability $c(n,g)$ that the lattice unit n is covered by a protein of type g and the probability $\theta(n,e)$ that the lattice unit is in chromatin state e are given as follows:

$$c(n,g) = \frac{\partial Z}{\partial K(n,g)} \times \frac{K(n,g)}{Z}; \theta(n,e) = \frac{\partial Z}{\partial s(n,e)} \times \frac{s(n,e)}{Z}. \quad (18)$$

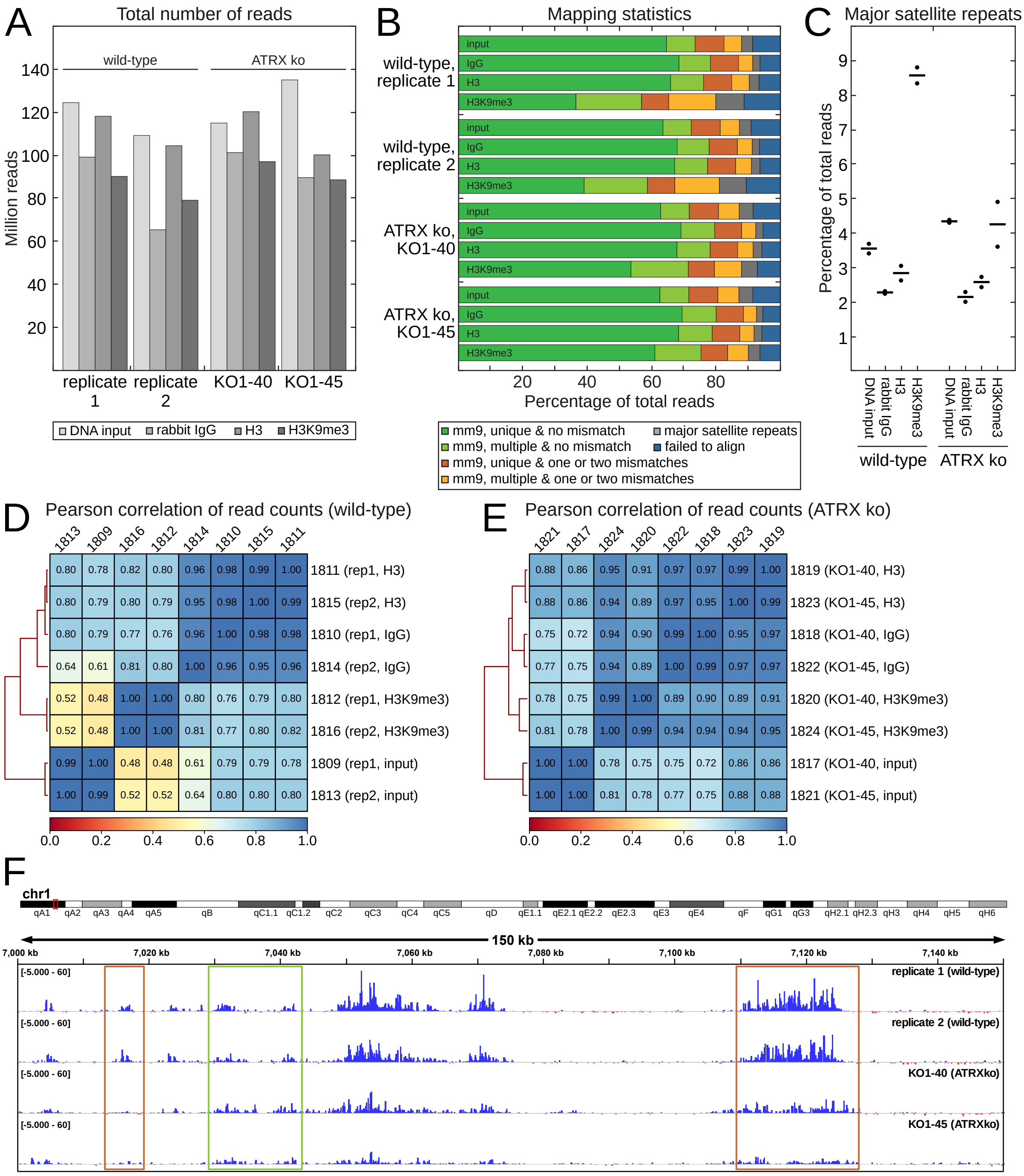

Supplementary Figure 1. Quality control and basic mapping features of the H3K9me3 ChIP-Seq data set for wild-type and ATRX knockout ESCs. (**A**) Total number of reads from sequencing on the Illumina HiSeq 2000 platform. (**B**) Basic mapping statistics using Bowtie2 [19]. Notably, reads from H3K9me3 IP tend to map to multiple sites in the genome (yellow, light green) as expected since repetitive sequences are known to frequently carry H3K9me3 [20]. (**C**) Reads mapping to major satellite repeats [21] by creating a dedicated Bowtie [22] index composed of 234 bp of the consensus repeat sequence and allowing up to two mismatches [23]. Major satellite repeats are known to be associated with H3K9me3 [24]. Notably, H3K9 trimethylation was reduced upon ATRX knockout in ESCs. The plot shows the average from

two ChIP-seq measurements for each condition marked by a line together with the two values indicated by dots. (**D**) Pearson correlation between read coverage of 10 kb bins throughout the genome calculated using deepTools2 [25] for wild-type samples. Importantly, replicates appear very similar to each other, and the H3K9me3 IPs were rather dissimilar from the input. (**E**) Same a panel D but for ATRX ko samples. These samples indicating showed a lower enrichment over input, which is likely to reflect some loss of H3K9me3. (**F**) IGV traces [26] of normalized and background-corrected H3K9me3 traces for both wild-type ESCs and upon ATRX knockout. Some regions appear rather independent of ATRX (green) whereas others showed less H3K9me3 enrichment in the ATRX ko samples compared to the wild-type (orange). Normalization and background correction were done as described previously using MCORE [27].

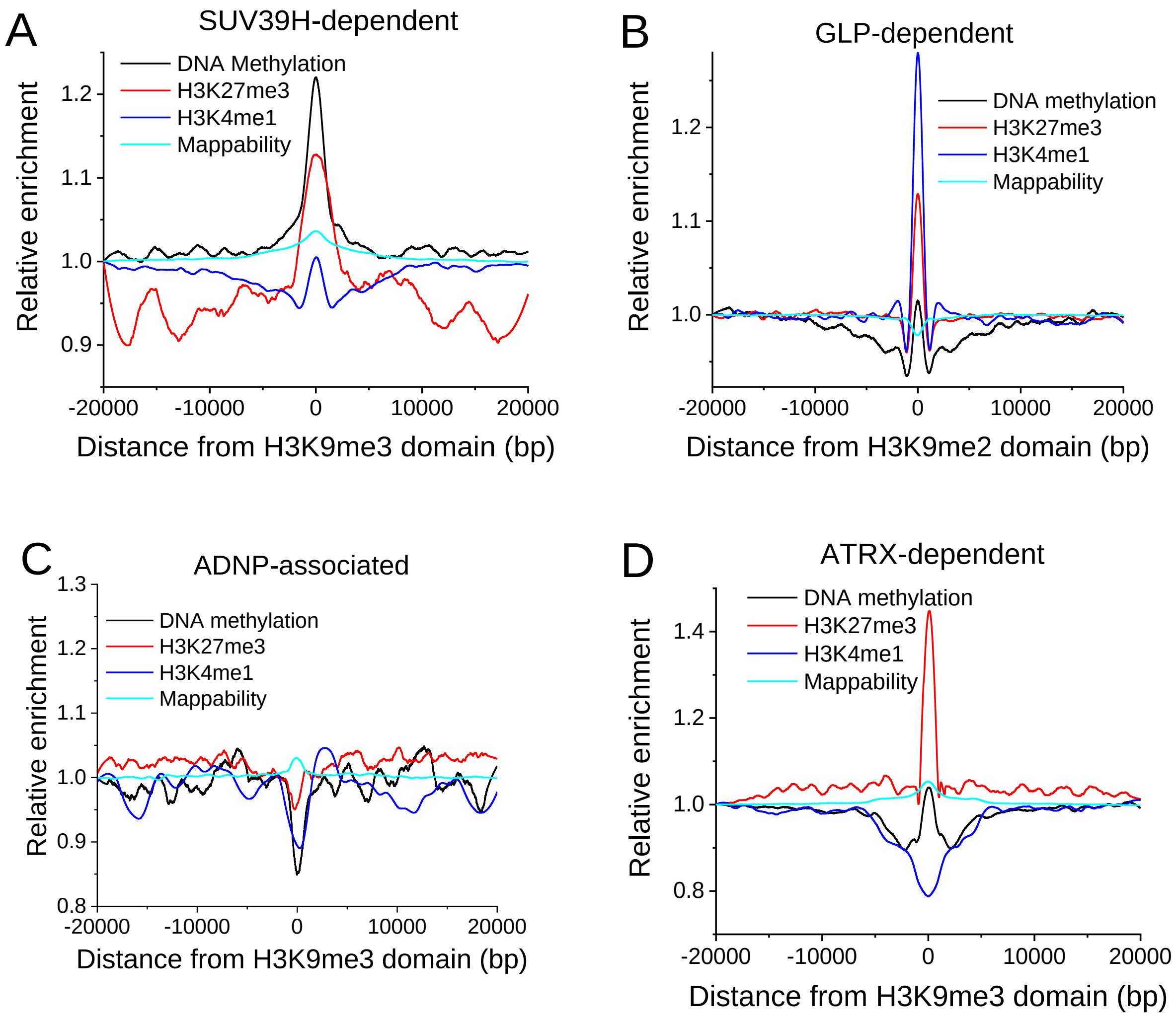

Supplementary Figure 2. Average profiles of DNA methylation density, H3K27me3, H3K4me1 and mappability across the four types of heterochromatin regions shown in this manuscript: (A) Suv39h dependent HNDs. (B) GLP-dependent HNDs. (C) ADNP-associated HNDs. (D) ATRX-dependent HNDs. Note than, in contrast to the other three sets of data, mappability for the GLP-dependent peaks falls in the centre, indicating a higher proportion of repeating genomic elements in this dataset.

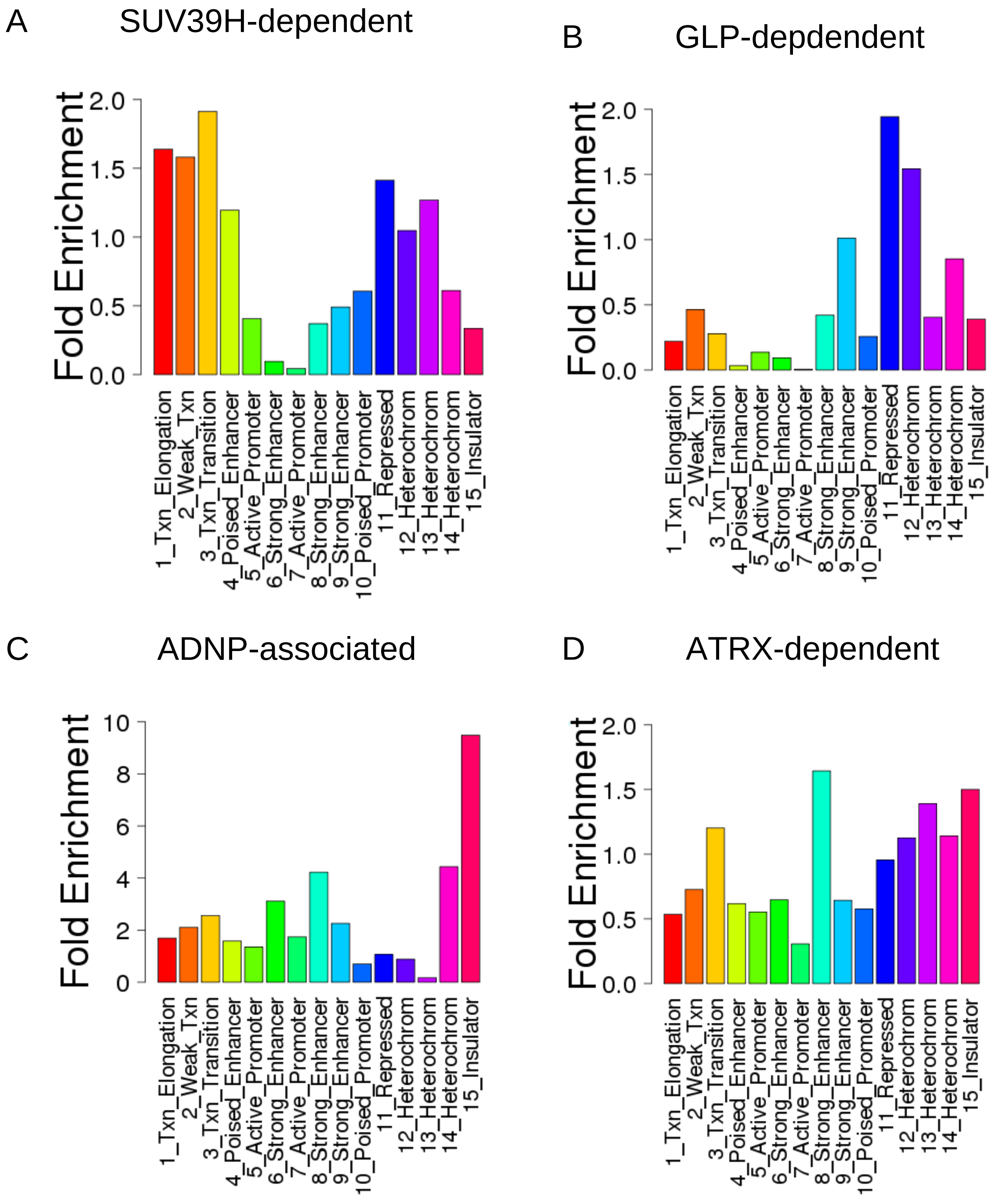

Supplementary Figure 3. Enrichment of ChromHMM-determined ESC states [28] in the sets of heterochromatin peaks used in this manuscript. (A) Suv39h-dependent HNDs. (B) GLP-dependent HNDs. (C) ADNP-associated HNDs. (D) ATRX-dependent HNDs.

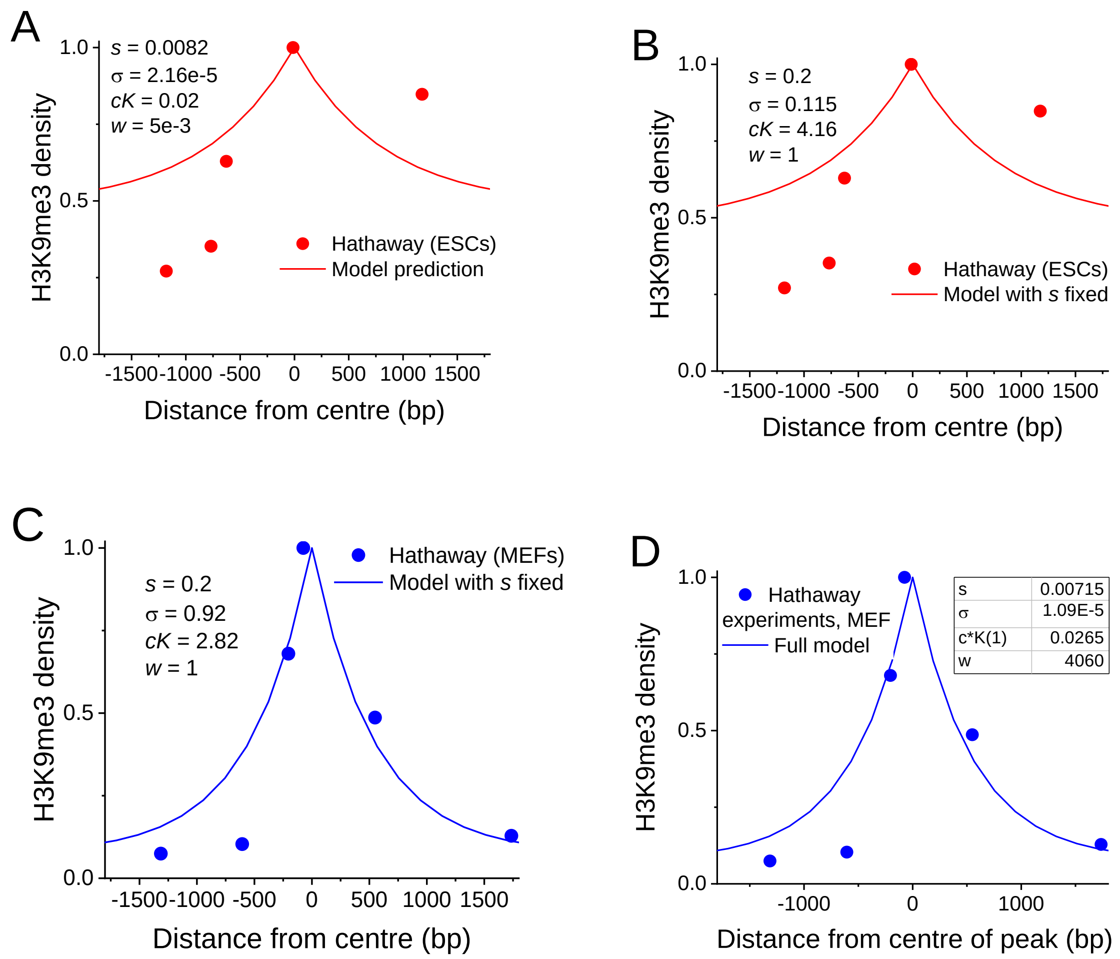

E

	s	σ	cK_1	W
ESC	8.18×10^{-3}	2.16×10^{-5}	2.02×10^{-2}	5.03×10^{3}
MEF	7.15×10^{-3}	1.09×10^{-5}	2.64×10^{-2}	4.06×10^{3}
Suv39h-dep	1.04×10^{-2}	1.46×10^{-1}	1.61×10^{-2}	5.11×10^{3}
Atrx-dep	4.29×10^{-1}	7.18×10^{-6}	2.55×10^{-2}	6.93×10^{2}
GLP-dep	7.10×10^{-3}	3.79×10^{-9}	2.37×10^{-2}	4.43×10^{3}
ESC (*s* fixed)	0.2 (fixed)	1.15×10^{-1}	4.16	1.00
MEF (*s* fixed)	0.2 (fixed)	9.19×10^{-2}	2.82	1.00

Supplementary Figure 4. Comparison of predicted heterochromatin profiles with those determined in artificial heterochromatin experiments of Hathaway et al (2012). (A) Artificially-induced heterochromatin in ESCs from Hathaway et al (2012) (full parameter search). (B) The same as in panel A but with fixed *s* parameter. (C) Artificially-induced heterochromatin in MEFs from Hathaway et al (2012) (fixed *s* parameter, cf. panel A. (D) Artificially-induced

heterochromatin in MEFs from Hathaway et al (2012) (full model). (E) Table of best fit parameters *s*, σ and w for the ESC and MEF data from Hathaway et al (2012), as well as the averaged profiles for Suv39h-dependent, ATRX-dependent and GLP-dependent HNDs in ESCs.

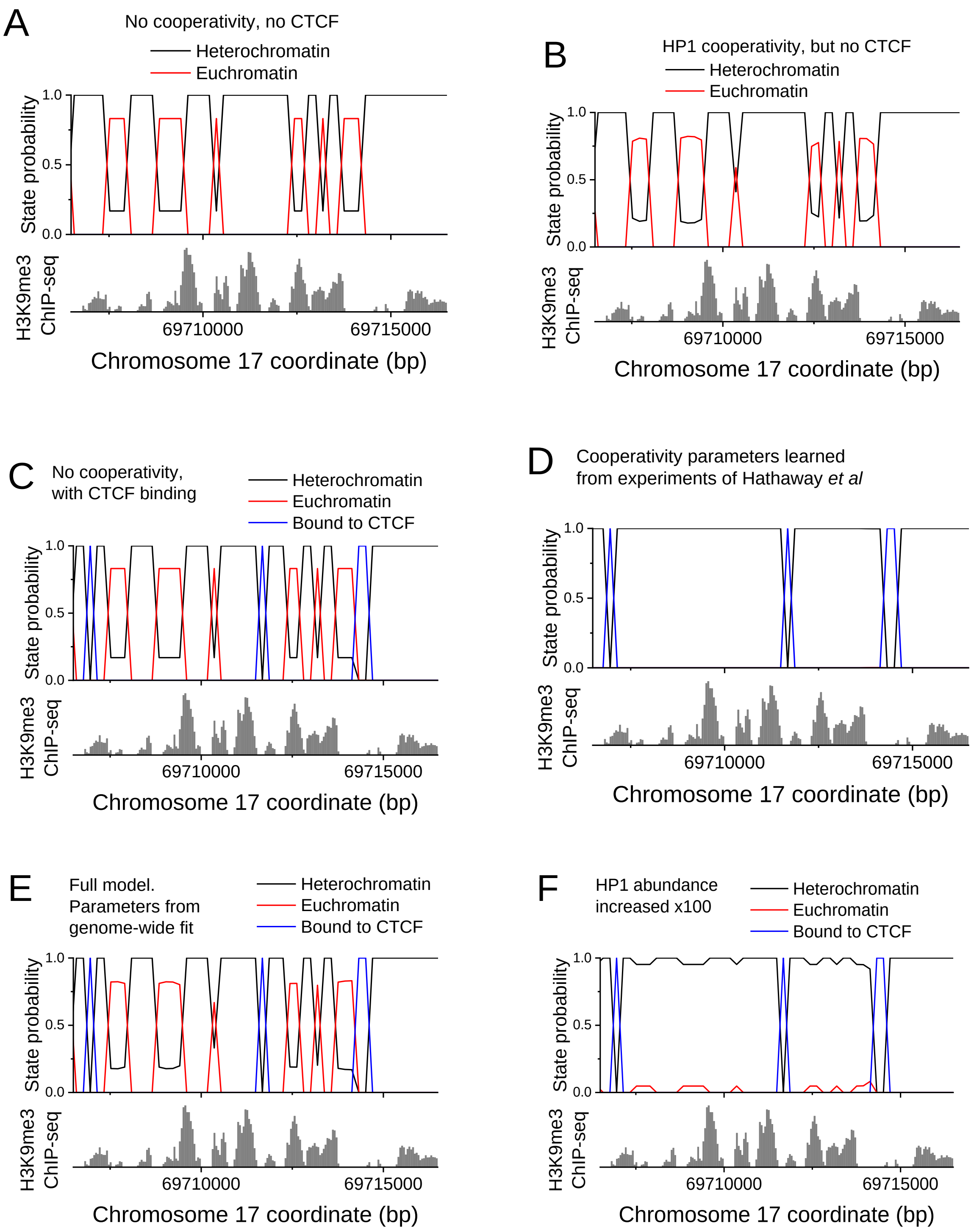

Supplementary Figure 5. Model predictions (top panels) and experimental H3K9me3 ChIP-Seq profiles (bottom panels) for an example region containing Suv39h-dependent HNDs. (A) No cooperativity of HP1 binding and no CTCF binding. (B) HP1 binding cooperativity but no CTCF binding. (C) CTCF binding but no HP1 cooperativity. (D) HP1 cooperativity, CTCF binding and nucleosome-nucleosome interactions (σ = 2.16e-5) learned from experiments of Hathaway *et al*). (E) Full model fitted to genome-wide *in vivo* ChIP-seq data in ESCs. F) Same as (E) but the local HP1 concentration is increased 100-fold.

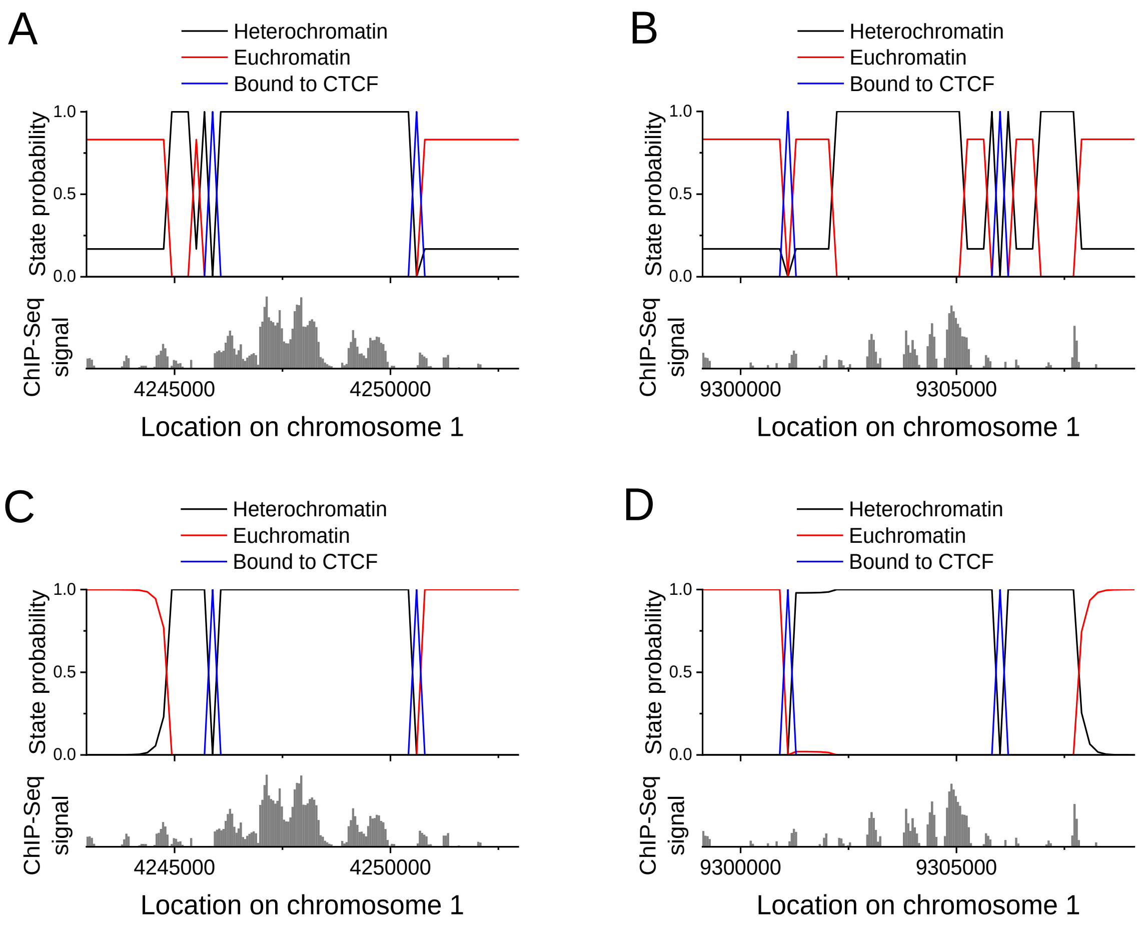

Supplementary Figure 6. The effect of CTCF removal exemplified for two regions containing Suv39-dependent HNDs, chr1:4242961-4252961 and chr1:9299120-9309120. (A) and (B) Predictions of the model using the best fit parameters from the Suv39-dependent heterochromatin peak size distribution, but without HP1-HP1 cooperativity. (C) and (D) predictions of the model using the parameters fitted to the Hathaway et al artificially established heterochromatin in MEF. The effect of HP1-HP1 cooperativity results in smoothing the heterochromatin propagation front and expanding the HND size.

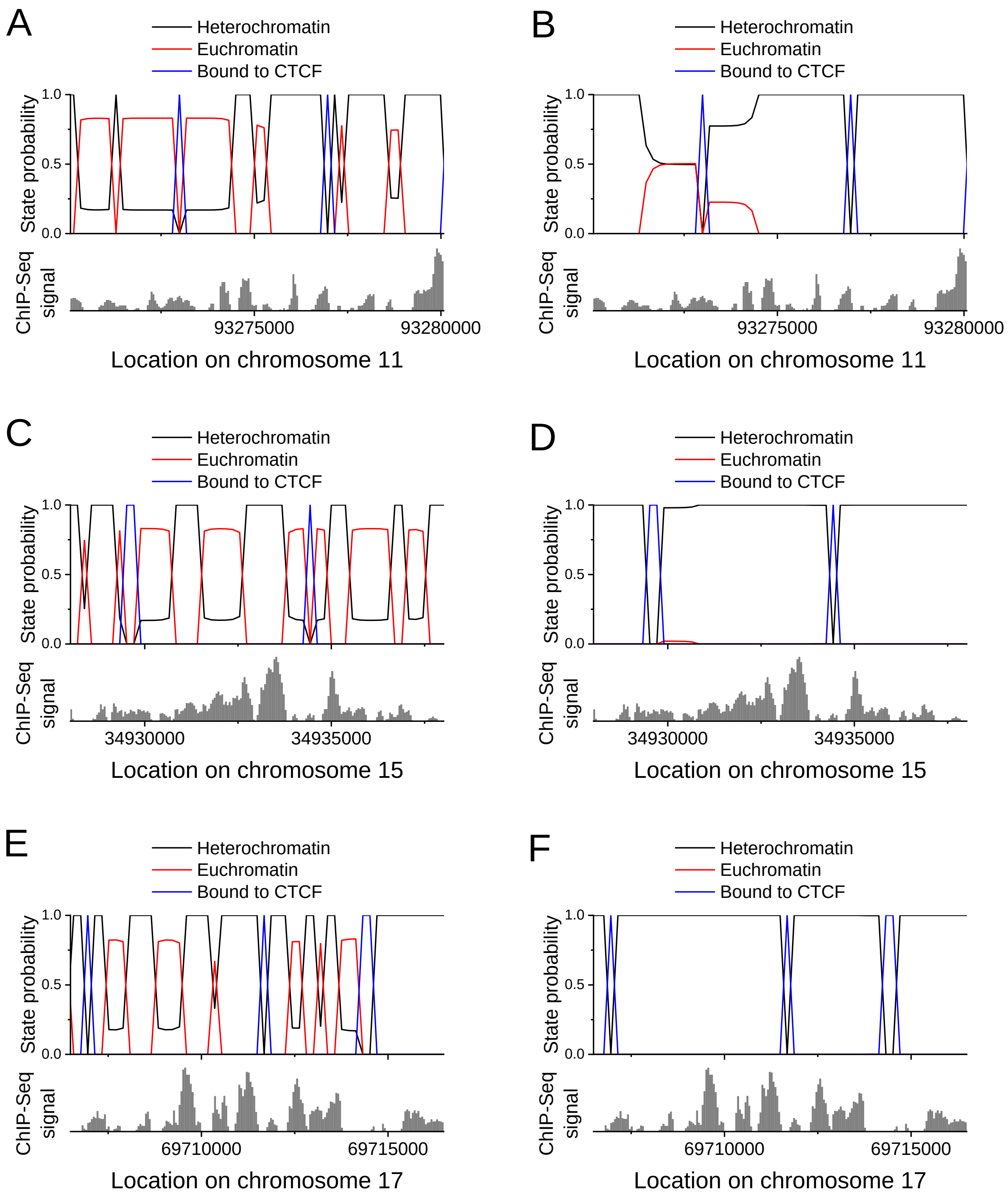

Supplementary Figure 7. The effect of reducing σ exemplified for three genomic regions containing several Suv39-dependent heterochromatin domains. Left-hand panels have been calculated for $\sigma = 1$; right-hand panels for $\sigma = 2.16 \times 10^{-5}$.

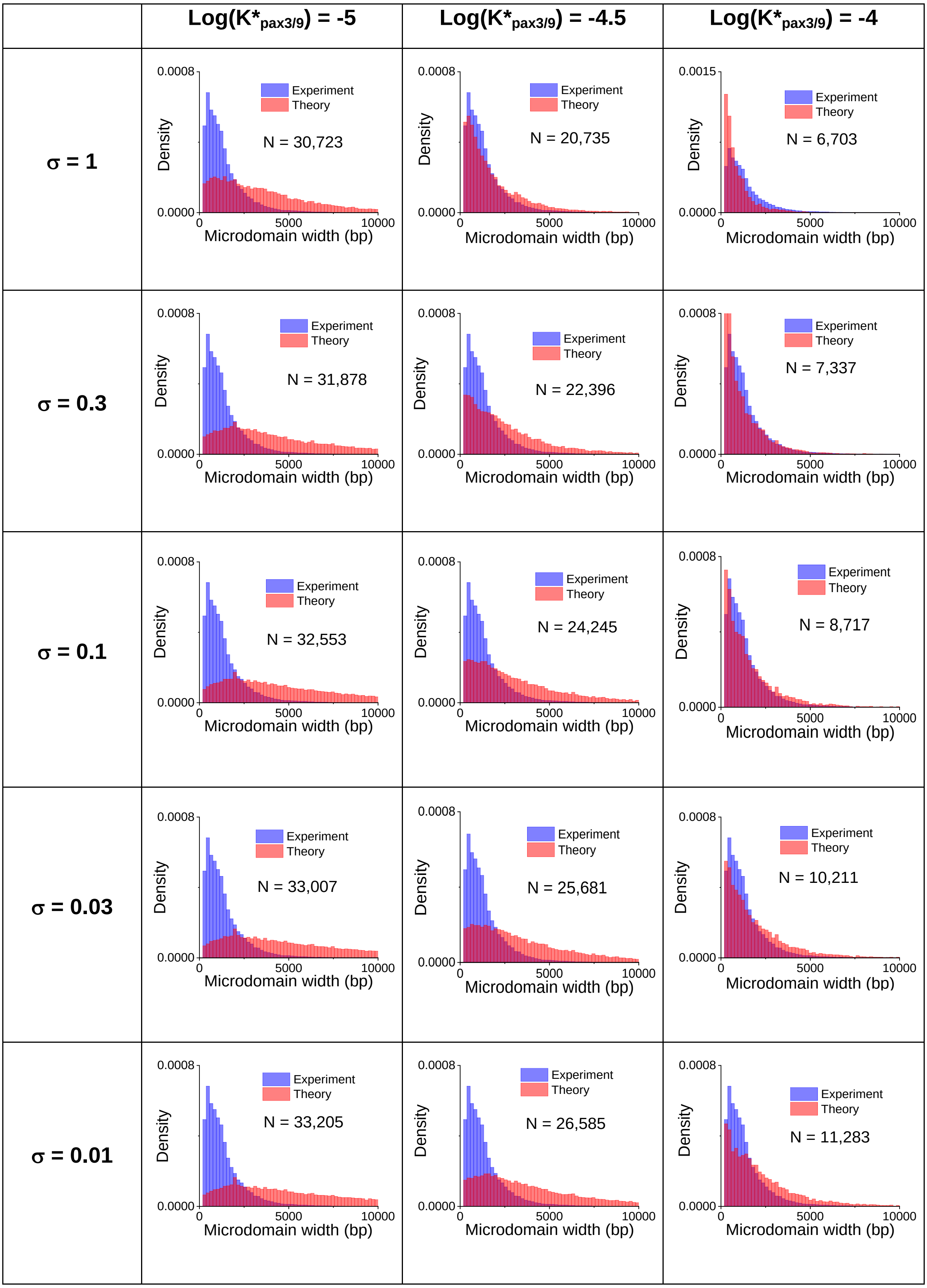

Supplementary Figure 8. Model predictions of Suv39h1/2-dependent nanodomain size distribution for varying values of the heterochromatin initiation threshold ($Log(K^*_{pax3/9})$) and σ (keeping other values fixed). Increasing either σ or $Log(K^*_{pax3/9})$ reduces the average HND size and the number of HNDs containing initiation sites. The best fit is in the top-middle cell.

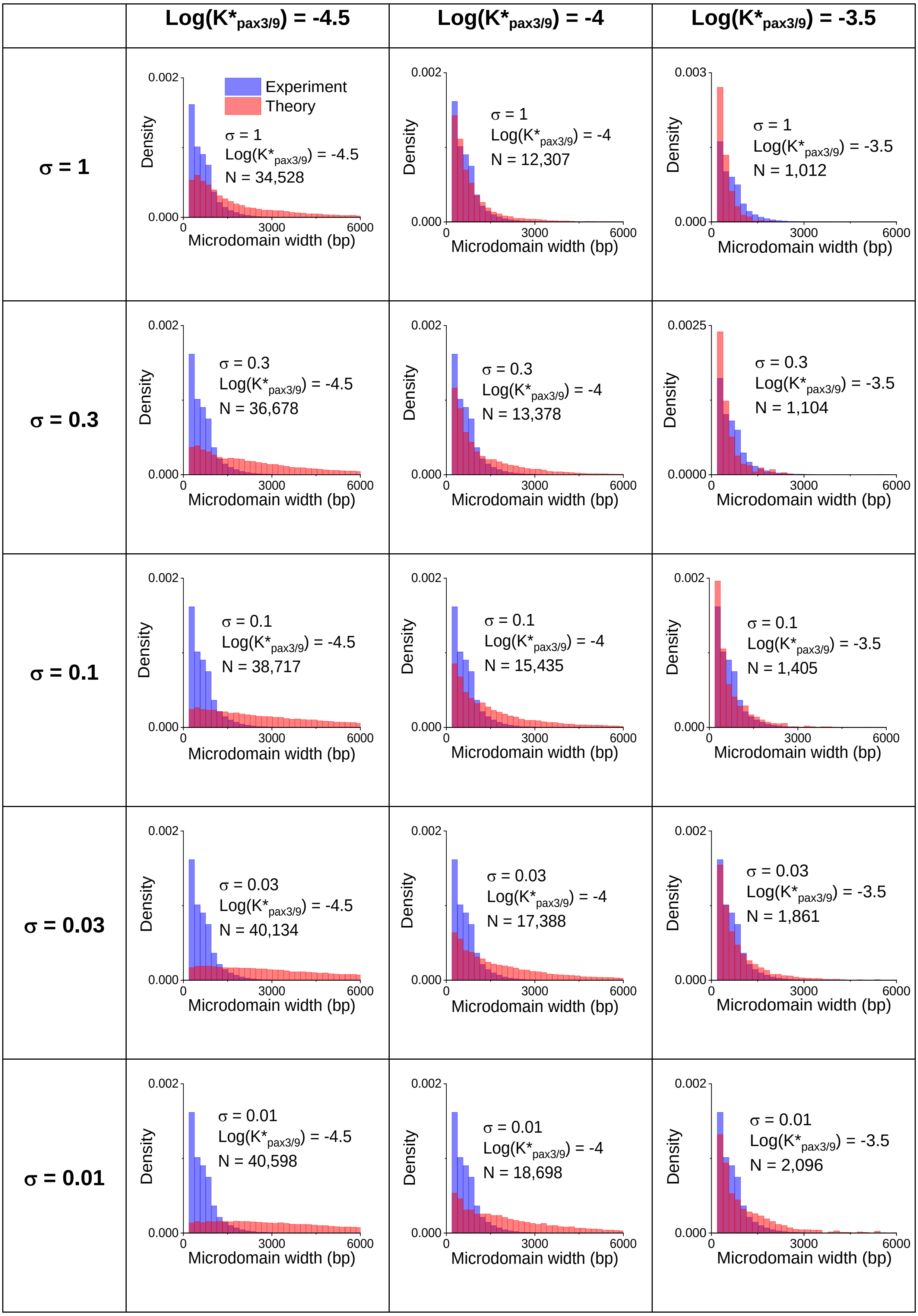
Log(K*pax3/9) = -4.5
Log(K*pax3/9) = -4
Log(K*pax3/9) = -3.5
σ = 1
σ = 0.3
σ = 0.1
σ = 0.03
σ = 0.01
Experiment
Theory
Density
Microdomain width (bp)
σ = 1 Log(K*pax3/9) = -4.5 N = 34,528
σ = 1 Log(K*pax3/9) = -4 N = 12,307
σ = 1 Log(K*pax3/9) = -3.5 N = 1,012
σ = 0.3 Log(K*pax3/9) = -4.5 N = 36,678
σ = 0.3 Log(K*pax3/9) = -4 N = 13,378
σ = 0.3 Log(K*pax3/9) = -3.5 N = 1,104
σ = 0.1 Log(K*pax3/9) = -4.5 N = 38,717
σ = 0.1 Log(K*pax3/9) = -4 N = 15,435
σ = 0.1 Log(K*pax3/9) = -3.5 N = 1,405
σ = 0.03 Log(K*pax3/9) = -4.5 N = 40,134
σ = 0.03 Log(K*pax3/9) = -4 N = 17,388
σ = 0.03 Log(K*pax3/9) = -3.5 N = 1,861
σ = 0.01 Log(K*pax3/9) = -4.5 N = 40,598
σ = 0.01 Log(K*pax3/9) = -4 N = 18,698
σ = 0.01 Log(K*pax3/9) = -3.5 N = 2,096

Supplementary Figure 9. Model predictions of GLP-dependent nanodomain size distribution for varying values of the heterochromatin initiation threshold (Log($K^*_{pax3/9}$)**)** and σ (keeping other values fixed). Increasing both σ and Log($K^*_{pax3/9}$) reduces the average size of nanodomains and the number of regions containing heterochromatin initiation sites. The parameters chosen for the main text are in the top-middle cell of this table.

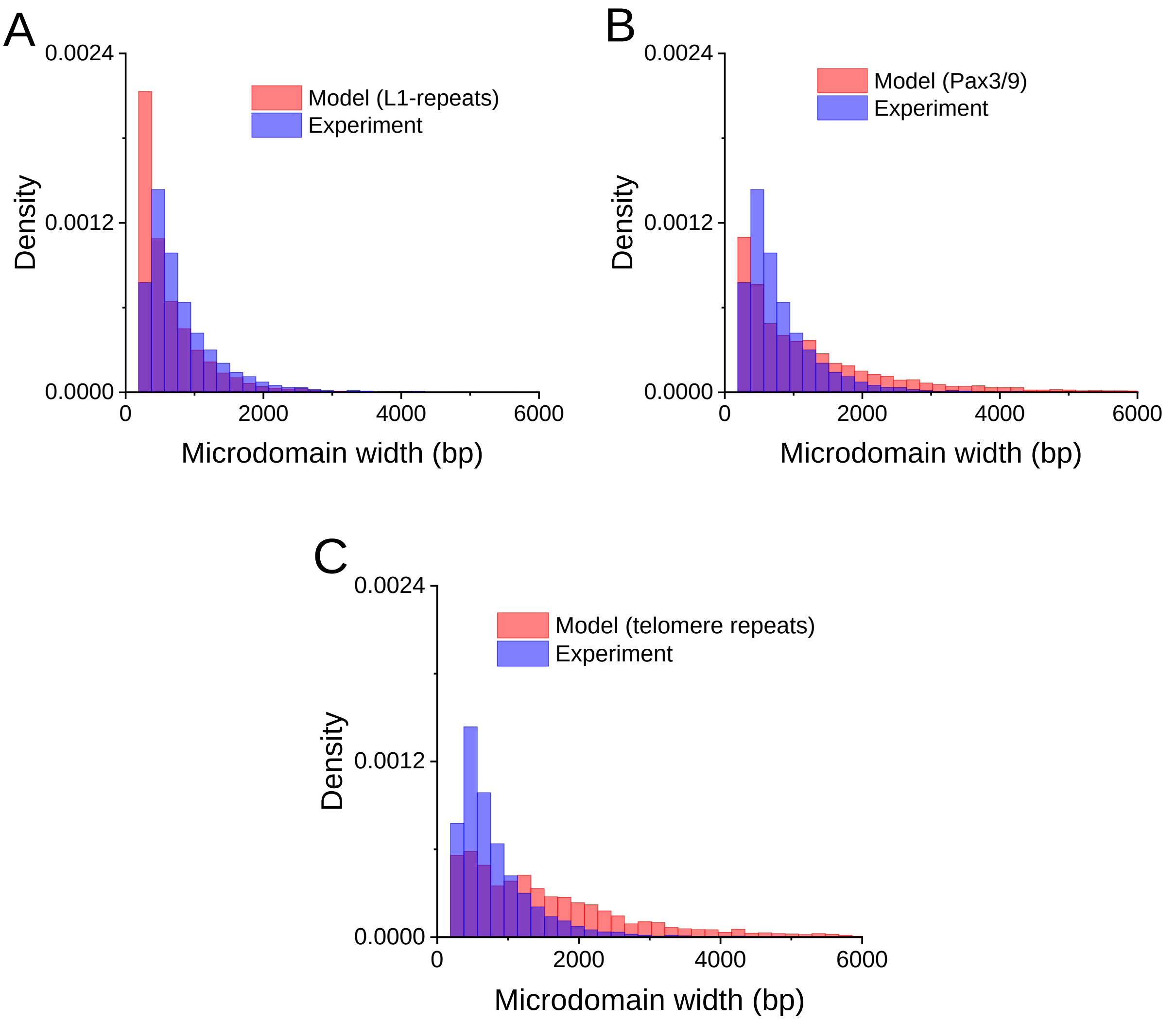

Supplementary Figure 10. Histograms of predicted peak distributions for the ATRX-dependent heterochromatin model using as initiation sites L1 repeats (A), PAX3/9 binding sites (B) and TERRA lncRNA-enriching telomere repeats (C).

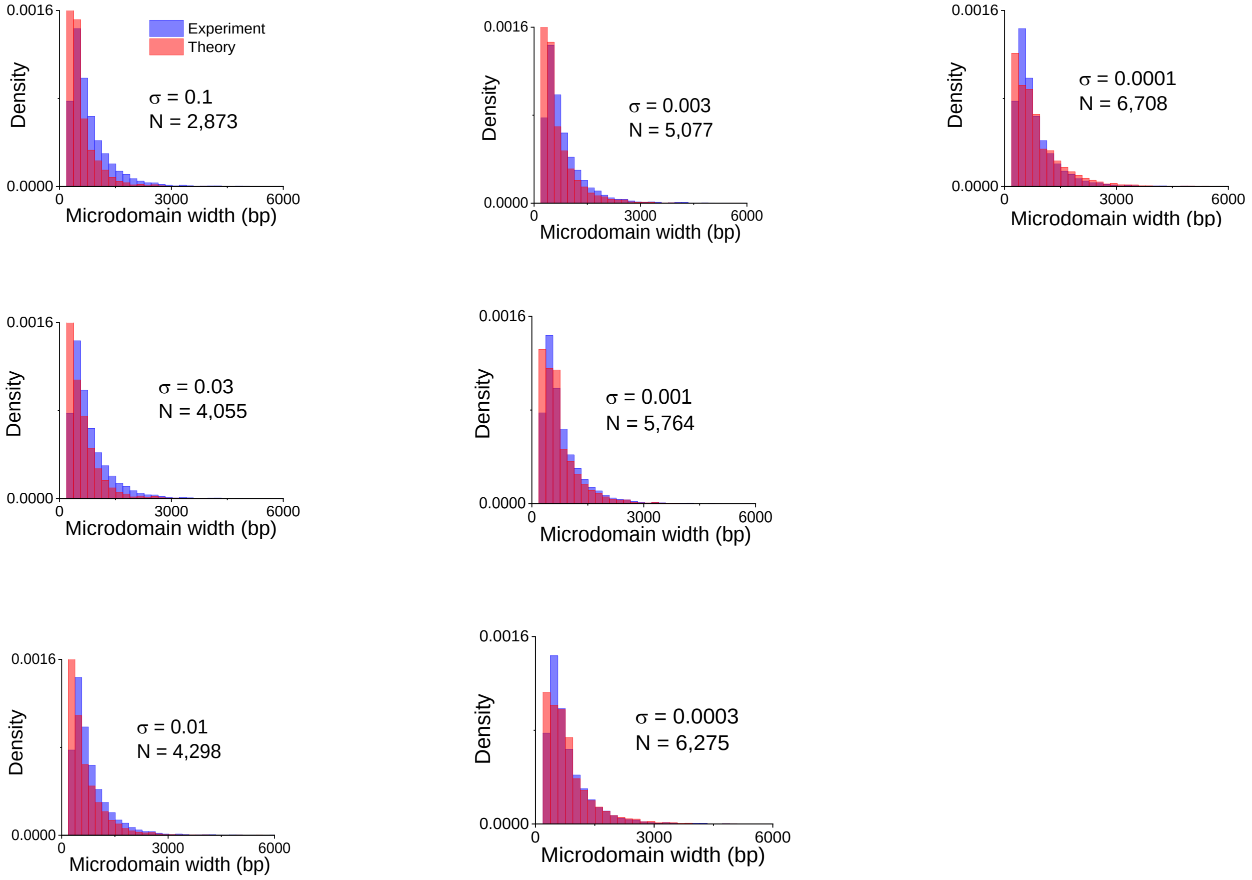

Supplementary Figure 11: Model predictions of ATRX-dependent nanodomain size distribution with experimental data for different choices of the boundary formation parameter σ using the L1 repeat-initiating model. Increasing σ reduces the size and number of peaks in the resulting distribution. In this case, heterochromatin is initiated in the model by the recognition of the repeat location, so the log-affinity threshold as it was in the Pax3/9-initiating model is not relevant. The choices of σ are indicated on the figure.

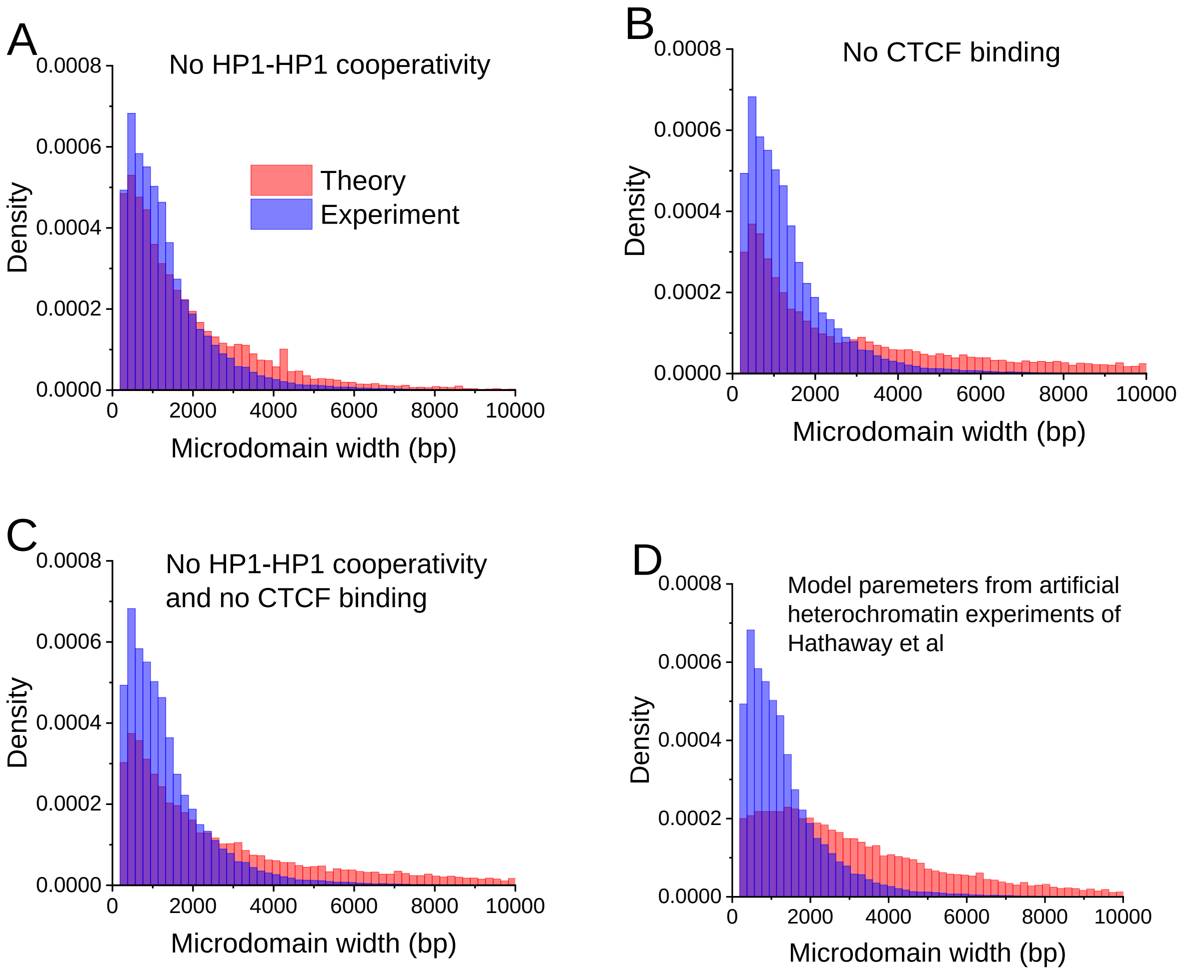

Supplementary Figure 12. The effects of HP1-HP1 cooperativity and CTCF binding on Suv39h-dependent heterochromatin formation. (A) Model without HP1-HP1 cooperativity. (B) Model without CTCF binding. (C) Model without CTCF binding and HP1-HP1 cooperativity. (D) Model using the parameters derived from the Hathaway et al [12] experiments for artificial heterochromatin establishment in ESCs.

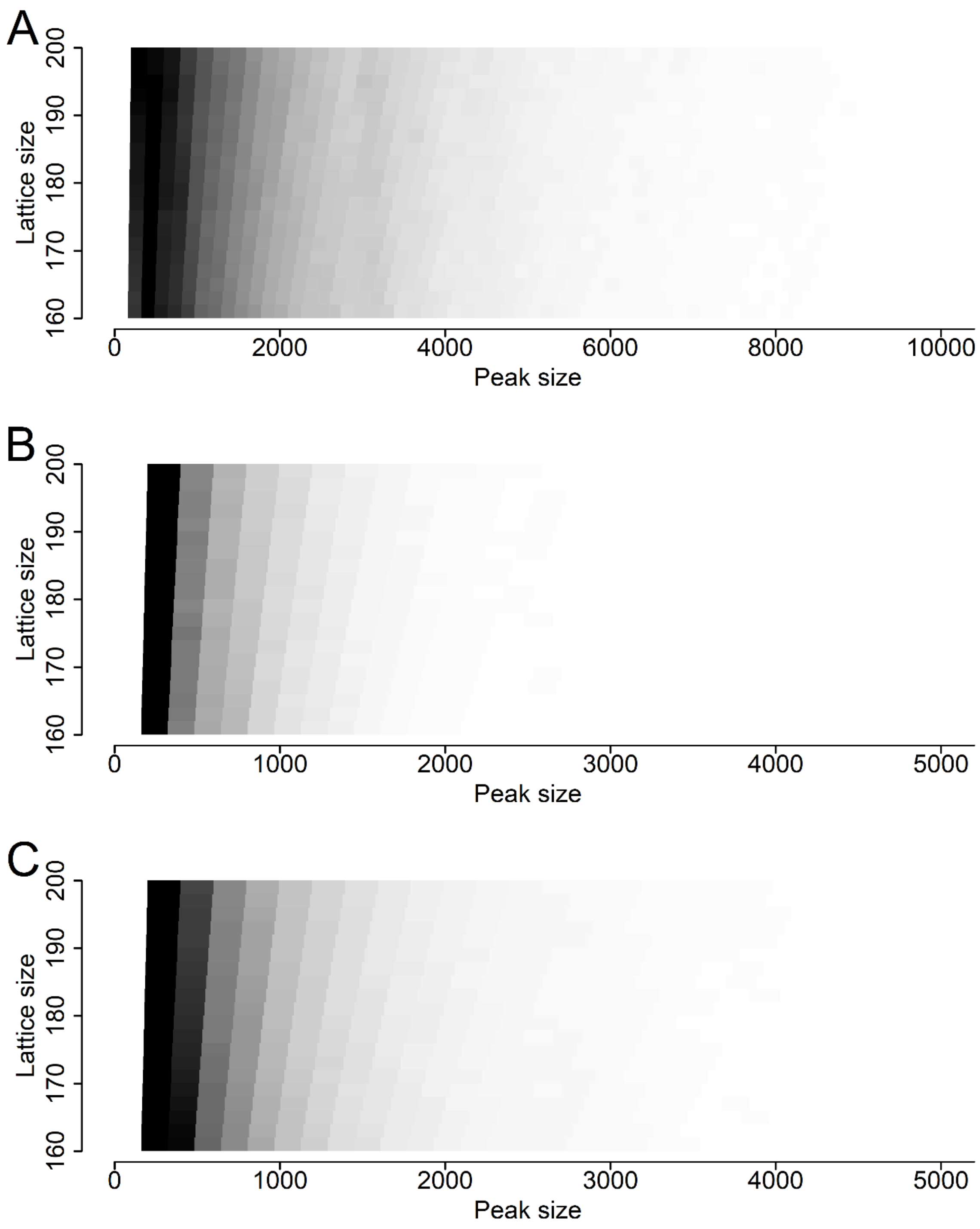
A
Lattice size
200
190
180
170
160
0
2000
4000
6000
8000
10000
Peak size
B
Lattice size
200
190
180
170
160
0
1000
2000
3000
4000
5000
Peak size
C
Lattice size
200
190
180
170
160
0
1000
2000
3000
4000
5000
Peak size

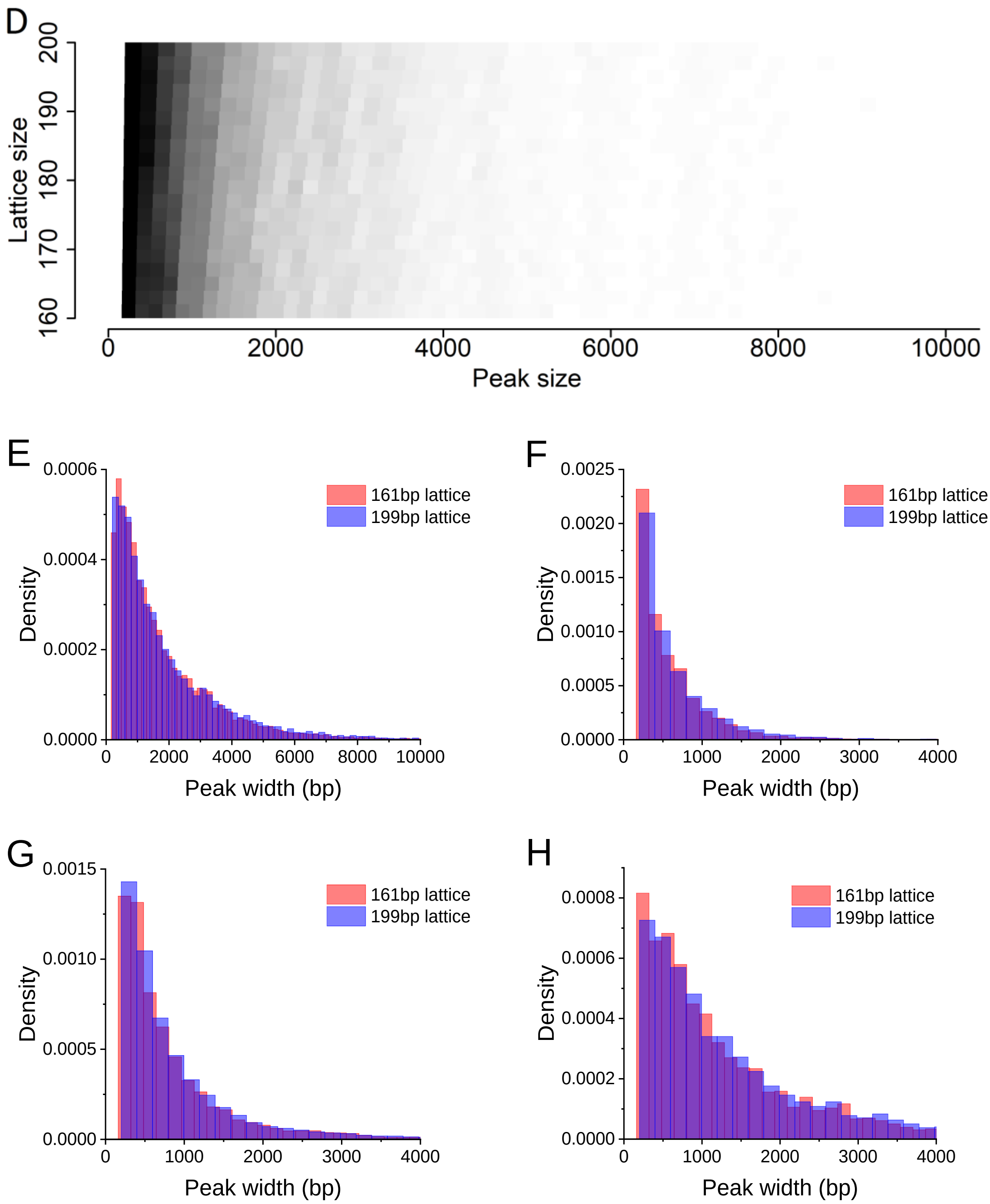

Supplementary Figure 13. Effect of the assumed chromatin lattice unit size (NRL) on the nanodomain size distribution. Darker areas indicate more peaks of that size in the distribution. (A) Suv39h-dependent heterochromatin. (B) ATRX-dependent heterochromatin. (C) GLP-dependent heterochromatin. (D) ADNP-dependent chromatin. (E-H) Comparisons of the predicted peak distributions for 161bp and 199bp lattice size for (E) Suv39h, (F) ATRX-dependent, (G) GLP-dependent HNDs and (H) ADNP-dependent HNDs.

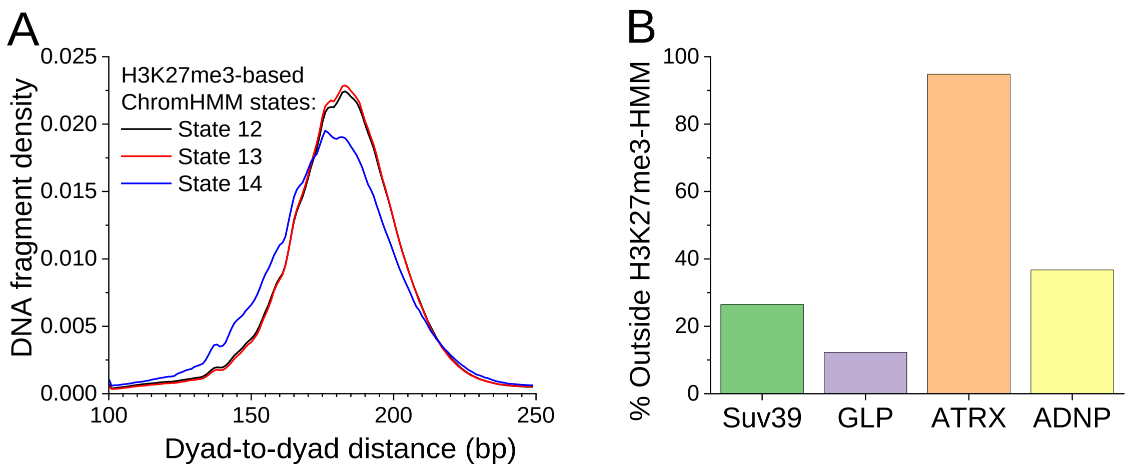

Supplementary Figure 14. (A) Nucleosome dyad-to-dyad distance distribution based on chemical mapping [29] for heterochromatin states determined using ChromHMM [28]. (B) Nucleosome dyad-to-dyad distance distributions for Suv39-, ATRX-, GLP-dependent HNDs that do not intersect with any of the three ChromHMM heterochromatin states.

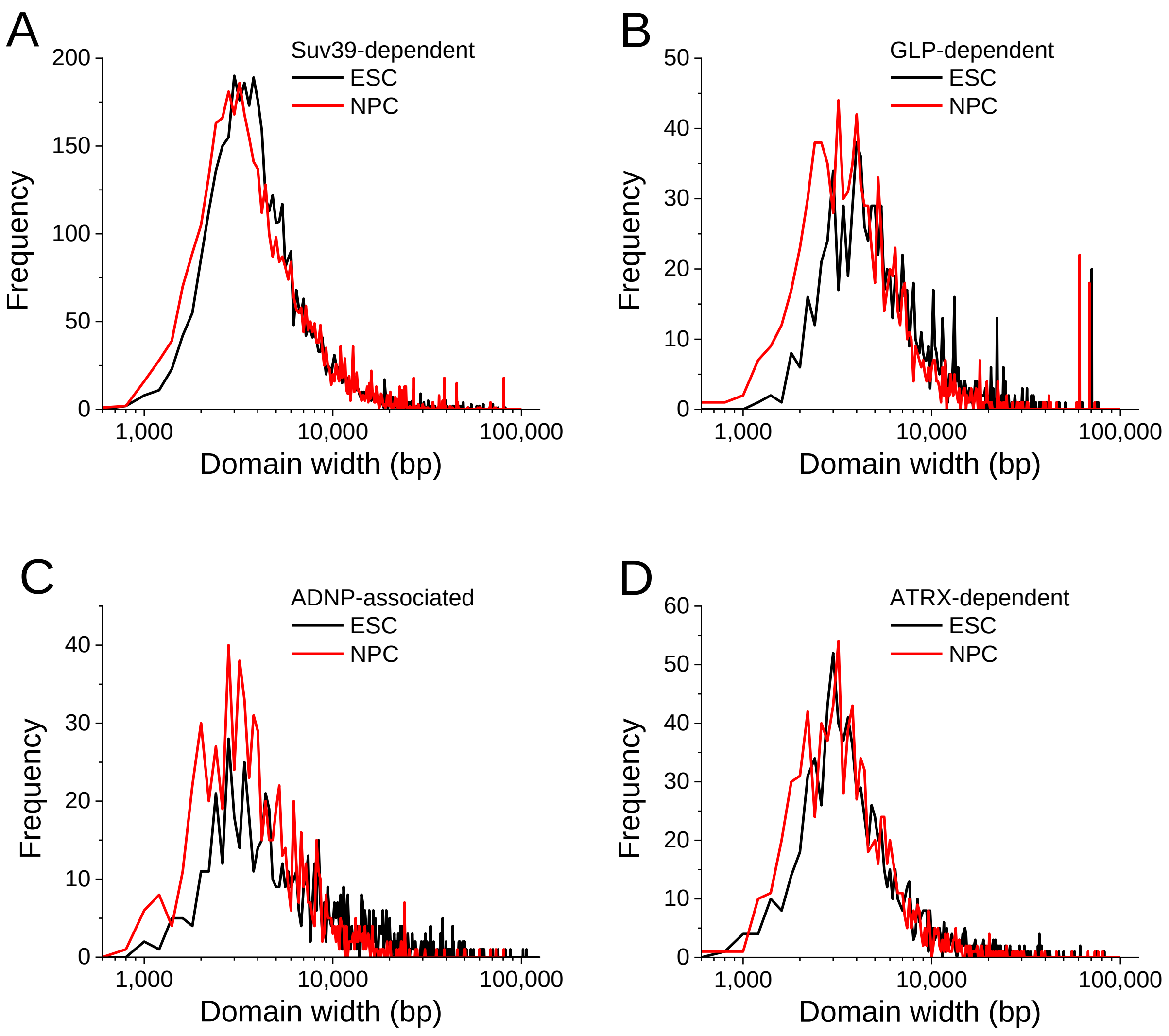

Supplementary Figure 15. Experimental distribution of HND sizes in ESCs vs. neural progenitor cells (NPCs) differentiated from them. Only heterochromatin regions which overlap between ESCs and NPCs were considered. (A) Suv39-dependent (n = 4510), (B) GLP-dependent (n = 1055), (C) ADNP-associated (n = 799), (D) ATRX-dependent H3K9-methylated domains (n = 961).

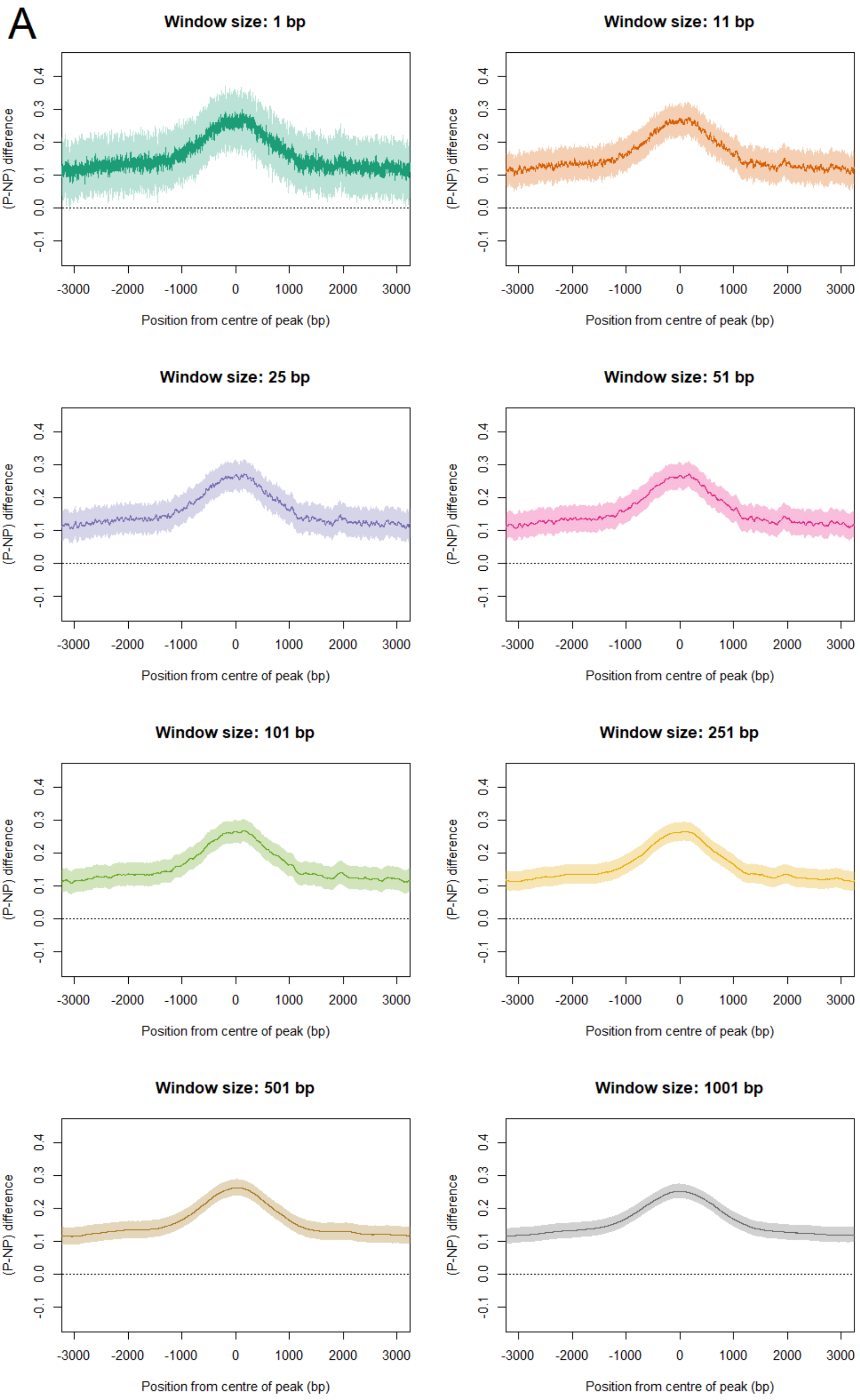
A
Window size: 1 bp
Window size: 11 bp
Window size: 25 bp
Window size: 51 bp
Window size: 101 bp
Window size: 251 bp
Window size: 501 bp
Window size: 1001 bp
(P-NP) difference
Position from centre of peak (bp)
-0.1
0.0
0.1
0.2
0.3
0.4
-3000
-2000
-1000
0
1000
2000
3000

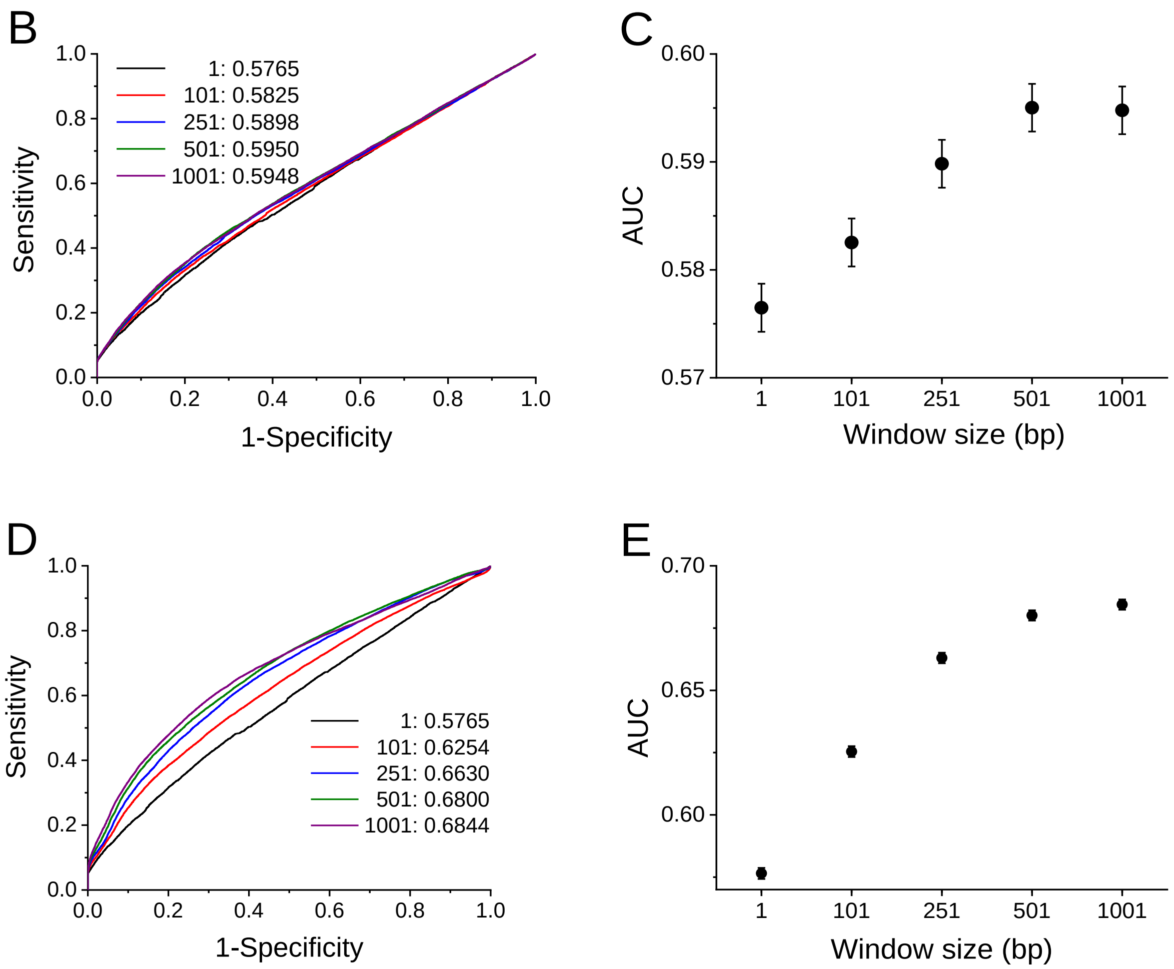

Supplementary Figure 16. TF-DNA binding affinity scores calculated using TRAP algorithm across Suv39h-dependent heterochromatin regions for different values of smoothing window: 1, 101, 251, 501 and 1001-bp centred geometrically averaged windows. (A) The difference between the logarithms of the affinity of TF-DNA binding geometrically averaged across ChIP-seq peaks (P) and non-peak regions (NP). The difference between the means of the logarithms of the affinity profiles for peaks and non-peaks is plotted, along with a band showing $\pm$ 5 times the sum of the standard errors for P and NP shown around the average line. (B) ROC curves based on the TRAP score with the matched non-peak regions, with AUCs for each window size shown. (C) Calculated AUCs for each window size with estimated standard error. (D) and (E) ROC and calculated AUC values using arithmetical smoothing for the same conditions as (B) and (C). In (C) and (E) the standard errors are computed with the integrated algorithm of ROC curve calculation in Origin Pro as detailed in the program manual (https://www.originlab.com/doc/en/Origin-Help/ROCCurve-Algorithm).

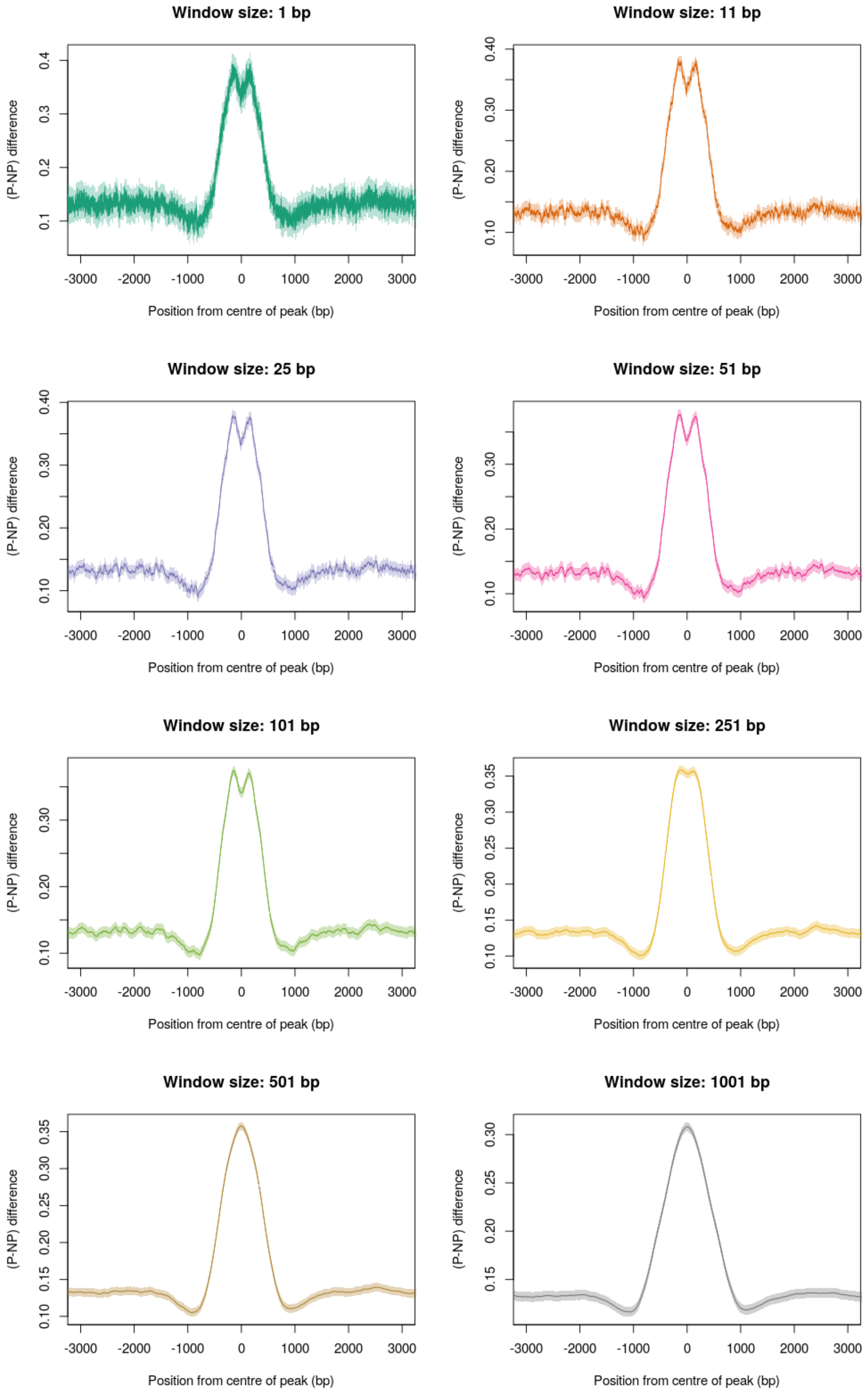

Supplementary Figure 17. TF-DNA binding affinity scores calculated using TRAP algorithm across GLP-dependent heterochromatin for different values of smoothing window. Values of 1, 101, 251, 501 and 1001-bp centred geometrically-averaged windows are shown. Similar to Figure S16, the difference between the logarithms of the affinity TF-DNA binding geometrically averaged across all peaks (P) non-peak regions (NP) as a solid line, and a band of $\pm$ 2 standard errors is shown on either side of the average.

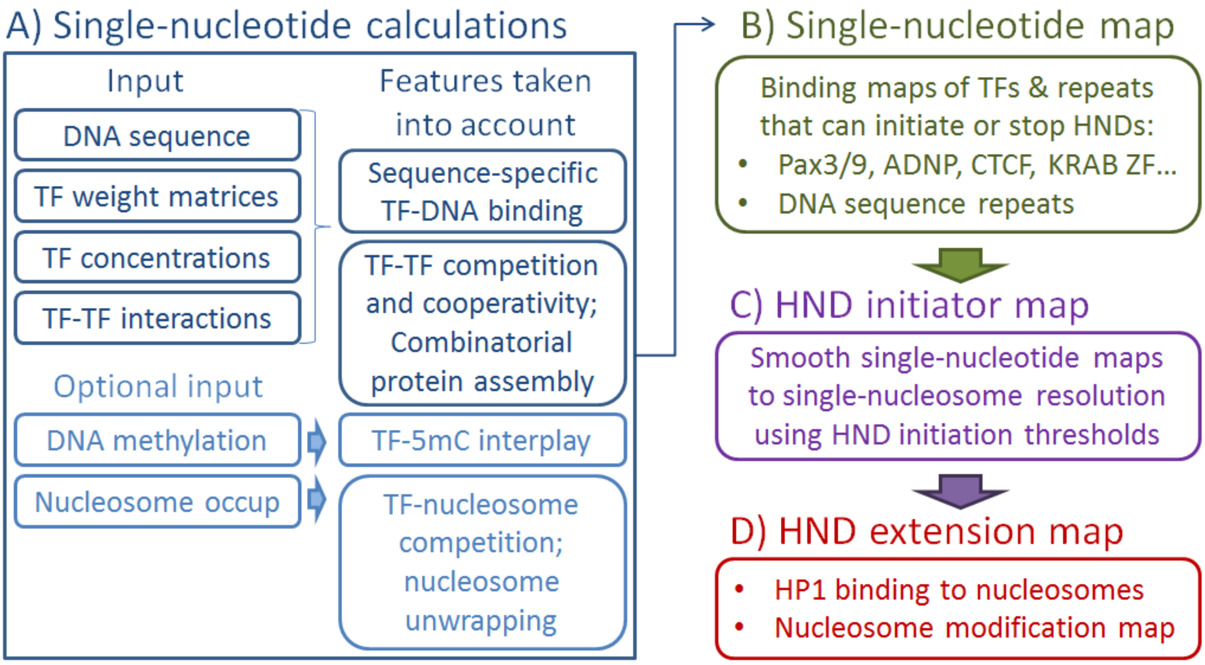

Supplementary Figure 18. ChromHL calculation workflow. (A) The input to the calculations with single-nucleotide resolution and the biophysical features taken into account. (B) The single-nucleotide resolution protein occupancies (binding maps) produced as a result step (A). (C) The coarse-graining of single-nucleotide resolution maps to arrive to the single-nucleosome resolution lattice; applying HND initiation thresholds to define elements of the nucleosome lattice that act as HND initiators. (D) Calculation with the nucleosome-resolution model to define the extension of HNDs and their boundaries.

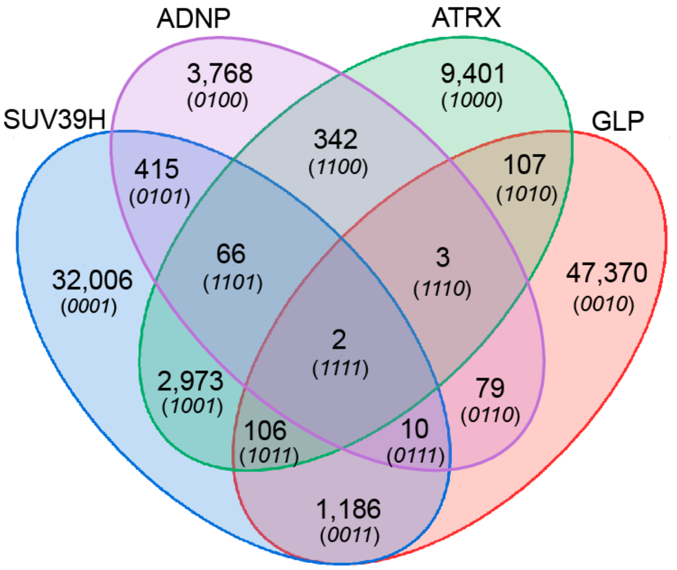

Supplementary Figure 19. Venn diagram showing intersections between different types of HNDs. The intersections are designated with a binary 1/0 code where "1" at position xxx1 corresponds to SUV39H HNDs, xx1x - GLP HNDs, x1xx - ADNP HNDs, 1xxx - ATRX HNDs.

Supplementary Table 1. Repeat motifs overlapping ATRX-dependent heterochromatin peaks.

Repeat name		Repeat class		Repeat family	
686	L1Md_F2	9483	LTR	5643	L1
668	L1Md_T	5853	LINE	4479	4ERVK
472	B3	3550	SINE	3002	MaLR
432	L1Md_A	2335	Simple_repeat	2335	Simple_repeat
408	MERVL-int	421	Low_complexity	1172	ERV1
335	ORR1D1	405	DNA	1075	Alu
321	B3A	139	Satellite	1054	B2
318	RLTR10	80	Other	980	B4
315	B4A	46	Unknown	827	ERVL
285	L1Md_F	34	rRNA	420	Low_complexity
275	Lx8	11	scRNA	313	MIR
249	ID_B1	10	snRNA	258	MER1_type
247	L1_Mus1	9	tRNA	173	L2
233	RSINE1	4	RNA	138	Satellite
231	(CA)n	2	srpRNA	124	ID
220	MTD	1	RC	80	Other
216	(TG)n			78	MER2_type
209	ETnERV2-int			42	Unknown
185	(TCTA)n			34	rRNA
183	B2_Mm2			32	CR1

The most frequently found repeats overlapping ATRX-dependent heterochromatin peaks are shown. Repeats were ordered by the repeat name, class of repeat and repeat family. Repeat annotations were obtained from the RepeatMasker track for Mus musculus (mm9) on the UCSC Genome Browser.

Supplementary Table 2. Chromatin features of different endogenous HND types

	SUV39H [a]	GLP [a]	ADNP [a]	ATRX [a]
**Marker modification**	H3K9me3	H3K9me2	H3K9me3	H3K9me3
**HND number**	36,764	48,881	4,673	13,113
**Typical HND size (kb)[b]**	2.0	0.9	1.1	0.7
**Nucleation motif**	PAX3/9	PAX3/9	ADNP	LINE1 subtype
**Correlation of HND size and the number of nucleation motifs [c]**	0.76	0.78	0.97	0.67
**HNDs with motif (%)**	92.4	95.9	100	100
**CTCF contribution to domain boundaries**	yes	yes	yes	no
**NRL**	189±1 bp	189±1 bp	175±1 bp	189±1 bp

[a] Protein factor upon which the HNDs are dependent in ESCs

[b] Domain size as described by the full width at half maximum of the aggregated H3K9me2/3 density plots shown in Fig. 3A-D

[c] Correlation coefficient between nanodomain size and the number of initiation motifs per domain.

Supplementary Table 3. Parameters of ChromHL models for the four types of HNDs in Figure 4.

	**SUV39H** [a]	**GLP** [a]	**ADNP** [a]	**ATRX** [a]
**Nucleation factor** [b]	PAX3/9	PAX3/9	ADNP	LINE1 binder
**HND initiation log threshold** [c]	-4.5	-4.0	-9.5	Any L1 repeat serves as initiation point
**s** [d]	0.2	0.2	0.2	0.2
**σ** [c]	1	1	1	0.01
***w* (HP1 cooperativity)** [e]	100	100	100	100

[a] Protein factor upon which the HNDs are dependent in ESCs.

[b] The nucleation factor is the chromatin protein that initiates HND formation.

[c] Log-affinity threshold for the binding probability of the nucleation factor that is enough to initiate HND formation. For ATRX-dependent HNDs, there is no threshold as the presence of any L1 repeat is enough to initiate HND in this model.

[d] In this calculation s was set at 0.2, like the latter fits in Figure S4. Fixing s at this value does not change the quality of the fit, as shown in that figure.

[e] This parameter was selected from the parameters fit from the Hathaway et al. fits (Figure S4). The resulting models are insensitive to the exact value of w, but not its order of magnitude. To simplify the fitting, w was fixed at 100

Supplementary Table 4. Position weight matrix (PWM) for ADNP based on 100-bp summits of 600 top ADNP-bound ChIP-seq peaks from Ostapcuk et al [15].

A	66	87	31	84	68	25	33	30	10	41	55	128	123	461
C	82	44	54	6	22	14	28	33	522	13	17	9	20	9
G	42	420	114	406	485	349	101	455	31	4	510	423	444	116
T	409	48	400	103	24	21	437	81	36	541	17	39	12	13

Supplementary References

1. Teif, V.B. & Rippe, K. Predicting nucleosome positions on the DNA: combining intrinsic sequence preferences and remodeler activities. *Nucleic Acids Res* **37**, 5641-5655 (2009).
2. Teif, V.B. General transfer matrix formalism to calculate DNA-protein-drug binding in gene regulation: application to O_R operator of phage λ. *Nucleic Acids Res* **35**, e80 (2007).
3. Teif, V.B. & Rippe, K. Statistical-mechanical lattice models for protein-DNA binding in chromatin. *J Phys Condens Matter* **22**, 414105 (2010).
4. Teif, V.B., Ettig, R. & Rippe, K. A lattice model for transcription factor access to nucleosomal DNA. *Biophys J* **99**, 2597-2607 (2010).
5. Teif, V.B., Kepper, N., Yserentant, K., Wedemann, G. & Rippe, K. Affinity, stoichiometry and cooperativity of heterochromatin protein 1 (HP1) binding to nucleosomal arrays. *J Phys Condens Matter* **27**, 064110 (2015).
6. Teif, V.B. & Rippe, K. Nucleosome mediated crosstalk between transcription factors at eukaryotic enhancers. *Phys Biol* **8**, 044001 (2011).
7. Ising, E. Beitrag zur Theorie des Ferromagnetismus. *Z. Phys.* **31**, 253-258 (1925).
8. Wartell, R.M. & Benight, A.S. Thermal denaturation of DNA molecules: A comparison of theory with experiment. *Physics Reports* **126**, 67-107 (1985).
9. McGhee, J.D. Theoretical calculations of the helix–coil transition of DNA in the presence of large, cooperatively binding ligands. *Biopolymers* **15**, 1345-1375 (1976).
10. Teif, V.B. et al. Taking into account nucleosomes for predicting gene expression. *Methods* **62**, 26-38 (2013).
11. Canzio, D. et al. Chromodomain-mediated oligomerization of HP1 suggests a nucleosome-bridging mechanism for heterochromatin assembly. *Mol Cell* **41**, 67-81 (2011).
12. Hathaway, N.A. et al. Dynamics and memory of heterochromatin in living cells. *Cell* **149**, 1447-1460 (2012).
13. Bulut-Karslioglu, A. et al. A transcription factor-based mechanism for mouse heterochromatin formation. *Nat Struct Mol Biol* **19**, 1023-1030 (2012).
14. Liu, N. et al. Recognition of H3K9 methylation by GLP is required for efficient establishment of H3K9 methylation, rapid target gene repression, and mouse viability. *Genes Dev* **29**, 379-393 (2015).
15. Ostapcuk, V. et al. Activity-dependent neuroprotective protein recruits HP1 and CHD4 to control lineage-specifying genes. *Nature* **557**, 739-743 (2018).
16. Teif, V.B. Ligand-induced DNA condensation: choosing the model. *Biophys J* **89**, 2574-87 (2005).
17. Teif, V.B., Haroutiunian, S.G., Vorob'ev, V.I. & Lando, D.Y. Short-range interactions and size of ligands bound to DNA strongly influence adsorptive phase transition caused by long-range interactions. *J Biomol Struct Dyn* **19**, 1093-1100 (2002).
18. Rippe, K. Liquid-Liquid Phase Separation in Chromatin. *Cold Spring Harb Perspect Biol* (2021).
19. Langmead, B. & Salzberg, S.L. Fast gapped-read alignment with Bowtie 2. *Nat Methods* **9**, 357-359 (2012).
20. Janssen, A., Colmenares, S.U. & Karpen, G.H. Heterochromatin: Guardian of the Genome. *Annu Rev Cell Dev Biol* **34**, 265-288 (2018).
21. Lehnertz, B. et al. Suv39h-Mediated Histone H3 Lysine 9 Methylation Directs DNA Methylation to Major Satellite Repeats at Pericentric Heterochromatin. *Current Biology* **13**, 1192-1200 (2003).
22. Langmead, B., Trapnell, C., Pop, M. & Salzberg, S.L. Ultrafast and memory-efficient alignment of short DNA sequences to the human genome. *Genome Biol* **10**, R25 (2009).
23. Beshnova, D.A., Cherstvy, A.G., Vainshtein, Y. & Teif, V.B. Regulation of the nucleosome repeat length in vivo by the DNA sequence, protein concentrations and long-range interactions. *PLoS Comput Biol* **10**, e1003698 (2014).

24. Peters, A.H. et al. Loss of the Suv39h histone methyltransferases impairs mammalian heterochromatin and genome stability. *Cell* **107**, 323-37 (2001).
25. Ramirez, F. et al. deepTools2: a next generation web server for deep-sequencing data analysis. *Nucleic Acids Res* **44**, W160-5 (2016).
26. Robinson, J.T. et al. Integrative genomics viewer. *Nat Biotechnol* **29**, 24-6 (2011).
27. Molitor, J., Mallm, J.P., Rippe, K. & Erdel, F. Retrieving Chromatin Patterns from Deep Sequencing Data Using Correlation Functions. *Biophys J* **112**, 473-490 (2017).
28. Bogu, G.K. et al. Chromatin and RNA Maps Reveal Regulatory Long Noncoding RNAs in Mouse. *Mol Cell Biol* **36**, 809-819 (2015).
29. Voong, L.N. et al. Insights into Nucleosome Organization in Mouse Embryonic Stem Cells through Chemical Mapping. *Cell* **167**, 1555-1570 (2016).
