# Supplementary material for: DNA sequence-dependent formation of heterochromatin nanodomains
Source: Nat Commun. 2022 Apr 6;13:1861. doi: 10.1038/s41467-022-29360-y (PMC8986797; doi:10.1038/s41467-022-29360-y)
Supplement: Supplementary file 2 — Reporting summary [file 41467_2022_29360_MOESM2_ESM.pdf]

nature research

Corresponding author(s): Vladimir B Teif and Karsten Rippe

Last updated by author(s): Feb 9, 2022

Reporting Summary

Nature Research wishes to improve the reproducibility of the work that we publish. This form provides structure for consistency and transparency in reporting. For further information on Nature Research policies, see our Editorial Policies and the Editorial Policy Checklist.

Statistics

For all statistical analyses, confirm that the following items are present in the figure legend, table legend, main text, or Methods section.

n/a	Confirmed	
☐	☒	The exact sample size (n) for each experimental group/condition, given as a discrete number and unit of measurement
☐	☒	A statement on whether measurements were taken from distinct samples or whether the same sample was measured repeatedly
☐	☒	The statistical test(s) used AND whether they are one- or two-sided<br>*Only common tests should be described solely by name; describe more complex techniques in the Methods section.*
☒	☐	A description of all covariates tested
☐	☒	A description of any assumptions or corrections, such as tests of normality and adjustment for multiple comparisons
☐	☒	A full description of the statistical parameters including central tendency (e.g. means) or other basic estimates (e.g. regression coefficient) AND variation (e.g. standard deviation) or associated estimates of uncertainty (e.g. confidence intervals)
☐	☒	For null hypothesis testing, the test statistic (e.g. F, t, r) with confidence intervals, effect sizes, degrees of freedom and P value noted<br>*Give P values as exact values whenever suitable.*
☒	☐	For Bayesian analysis, information on the choice of priors and Markov chain Monte Carlo settings
☐	☒	For hierarchical and complex designs, identification of the appropriate level for tests and full reporting of outcomes
☐	☒	Estimates of effect sizes (e.g. Cohen's d, Pearson's r), indicating how they were calculated

Our web collection on statistics for biologists contains articles on many of the points above.

Software and code

Policy information about availability of computer code

Data collection	ATRX ChIP-seq libraries were generated with the NEBNext Ultra DNA Library Prep Kit for Illumina (New England Biolabs, NEB #E7370). Sequencing was done on the Illumina HiSeq 2000 platform. Alignment of sequencing reads was conducted with the Bowtie2 software allowing for up to 2 mismatches software (bowtie-bio.sourceforge.net/bowtie2/index.shtml)
Data analysis	Manipulations with BED files were performed using bedTools 2.18 (https://github.com/arq5x/bedtools2).<br>Telomeric repeats were defined using RSAT 2018 (http://rsat.sb-roscoff.fr/).<br>G-quadruplex repeats were defined with fastaRegexFinder (https://github.com/dariober/bioinformatics-cafe/tree/master/fastaRegexFinder).<br>PWM for sequence-specific ADNP binding using MEME 5.3.0 (http://meme-suite.org/tools/meme-chip).<br>Peaks were called with MACS2 (https://pypi.org/project/MACS2/) and EPIC 0.2.12 (https://github.com/biocore-ntnu/epic).<br>The nucleosome repeat length (NRL) was determined using NucTools 2 (https://homeveg.github.io/nuctools/).<br>The ChromHL software developed in this manuscript is available at https://github.com/TeifLab/ChromHL.<br>Data visualisation was done with Origin Pro 2019, Origin Pro 2020 and Origin Pro 2021 (https://www.originlab.com/)

For manuscripts utilizing custom algorithms or software that are central to the research but not yet described in published literature, software must be made available to editors and reviewers. We strongly encourage code deposition in a community repository (e.g. GitHub). See the Nature Research guidelines for submitting code & software for further information.

Data

Policy information about availability of data

All manuscripts must include a data availability statement. This statement should provide the following information, where applicable:

- Accession codes, unique identifiers, or web links for publicly available datasets
- A list of figures that have associated raw data
- A description of any restrictions on data availability

Data from the ATRX knockout experiments generated in this study is deposited in the GEO database under accession number GSE158744. Previously published datasets used in our analysis are available in GEO entries GSE40086, GSE54412, GSE97945, GSE61874, GSE57092, GSE40910, GSE82127, GSE40910, GSE29184, GSE57092 and GSE30206 as detailed below.

For a given HND type, differential H3K9me3 or H3K9me2 peaks were called with MACS2 based on the WT and KO datasets of Suv39h1/h2 (GSE40086), Glp (GSE54412), and ATRX (GSE158744). ADNP-associated HNDs were defined as the intersection of ADNP-bound ChIP-seq peaks with all H3K9me3 peaks in wild type ESCs from these experiments (GSE97945). For H3K9me3 in NPCs, we used datasets GSE61874 and GSE57092 with peak calling performed by MACS for Fig. S15 as well as EPIC for Fig. 6B. The nucleosome repeat length (NRL) was determined using NucTools based on the previously published MNase-seq dataset (GSE40910). The dyad-dyad differences were computed using the chemical mapping dataset (GSE82127). In addition, we used published ChIP-seq datasets of CTCF (GSE29184) and HP1 (GSE57092), CpG methylation (GSE30206), and H3K4me1 and H3K27me3 histone modifications (GSE29184).

Field-specific reporting

Please select the one below that is the best fit for your research. If you are not sure, read the appropriate sections before making your selection.

☒ Life sciences ☐ Behavioural & social sciences ☐ Ecological, evolutionary & environmental sciences

For a reference copy of the document with all sections, see nature.com/documents/nr-reporting-summary-flat.pdf

Life sciences study design

All studies must disclose on these points even when the disclosure is negative.

Sample size	For the ATRX ChIP-seq, two independently generated samples were analyzed for each of the four sample types (input, IgG, H3 and H3K9me3) for both wildtype and ATRX knockout cells.
Data exclusions	No data were excluded from the analysis.
Replication	All eight different ATRX ChIP-seq readouts were repeated once. Replication was successful as shown in Supplementary Figure S1.
Randomization	No randomization was used. The analysis of ATRX by ChIP-seq was observational and studied the effect of the ATRAX knock-out.
Blinding	Investigators were not blinded during data collection and analysis.

Reporting for specific materials, systems and methods

We require information from authors about some types of materials, experimental systems and methods used in many studies. Here, indicate whether each material, system or method listed is relevant to your study. If you are not sure if a list item applies to your research, read the appropriate section before selecting a response.

Materials & experimental systems

n/a	Involved in the study	
☐	☒	Antibodies
☐	☒	Eukaryotic cell lines
☒	☐	Palaeontology and archaeology
☒	☐	Animals and other organisms
☒	☐	Human research participants
☒	☐	Clinical data
☒	☐	Dual use research of concern

Methods

n/a	Involved in the study	
☐	☒	ChIP-seq
☒	☐	Flow cytometry
☒	☐	MRI-based neuroimaging

Antibodies

Antibodies used	Anti-H3K9me3 antibody (Abcam, ab8898, lot: GR148830-2); Rabbit IgG antibody (R&D Systems, AB-105-C, lot: ER1212071); H3 rabbit polyclonal antibody (Abcam, ab179, lot: GR103864-1)
Validation	Both antibodies used in this study are commercially available with validation procedures described on the following sites of the

Validation

manufacturers:
H3K9me3 (Abcam), https://www.abcam.com/histone-h3-tri-methyl-k9-antibody-chip-grade-ab8898.html, ChIP-grade and validated by the manufacturer for ChIP analysis and previously used in the publications listed at the web page.
Rabbit IgG (R&D Systems), https://www.rndsystems.com/products/normal-rabbit-igg-control_ab-105-c#product-citations, control for unspecific binding, validated by the manufacturer and previously used in the publications listed at the web page.
H3 rabbit polyclonal (ChIP-grade) from Abcam (ab179, lot GR103864-1), https://www.abcam.com/histone-h3-antibody-nuclear-marker-and-chip-grade-ab1791.html, validated by the manufacturer for ChIP analysis and previously used in the publications listed at the web page.

Eukaryotic cell lines

Policy information about cell lines

Cell line source(s)

Wild type murine embryonic stem cells (ESCs) wt26 and Atrx knock out cell lines (KO1-40 and KO1-45) were described in our previous publication (Sadic, D. et al. Atrx promotes heterochromatin formation at retrotransposons. EMBO Rep 16, 836-50 (2015)).

Authentication

The cells lines studied by ATRX ChIP-seq were generated and characterized in a previous study (Sadic et al. Atrx promotes heterochromatin formation at retrotransposons. EMBO Rep 16, 836-50, 2015). Their authenticity was validated by comparing the sequencing data generated in the present work with those from the Sadic et al. study.

Mycoplasma contamination

All cell lines were tested for the absence of mycoplasma with the VenorGeM Advance kit (Minerva Biolabs, Berlin, Germany),

Commonly misidentified lines
(See ICLAC register)

The study does not use commonly misidentified cell lines.

ChIP-seq

Data deposition

☒ Confirm that both raw and final processed data have been deposited in a public database such as GEO.

☒ Confirm that you have deposited or provided access to graph files (e.g. BED files) for the called peaks.

Data access links
May remain private before publication.

The original sequencing data generated in this study are publicly available at Gene Expression Omnibus (GEO) under accession number GSE158744 (https://www.ncbi.nlm.nih.gov/geo/query/acc.cgi?acc=GSE158744)

Files in database submission

Processed file:
GSE158744_Atrx_peaks.bed
Raw data:
GSM4809449 H3K9me3 ChIP-seq WT rep1
GSM4809450 H3K9me3 ChIP-seq WT rep2
GSM4809451 H3K9me3 ChIP-seq ATRX KO rep1
GSM4809452 H3K9me3 ChIP-seq ATRX KO rep2
GSM4809453 Input WT rep1
GSM4809454 Input WT rep2
GSM4809455 Input ATRX KO rep1
GSM4809456 Input ATRX KO rep2
GSM5844772 IgG WT rep1
GSM5844773 IgG WT rep2
GSM5844774 IgG ATRX KO rep1
GSM5844775 IgG ATRX KO rep2
GSM5844776 H3 WT rep1
GSM5844777 H3 WT rep2
GSM5844778 H3 ATRX KO rep1
GSM5844779 H3 ATRX KO rep2

Genome browser session
(e.g. UCSC)

https://genome.ucsc.edu/s/vteif%40essex.ac.uk/ChromHL_v5

Methodology

Replicates

ChIP-seq and the sequencing of the corresponding Input samples was performed with two replicates in each of the two conditions (wild-type and ATRX knockout)

Sequencing depth

All samples were sequenced with 50-bp single-end reads, as detailed below:

Input WT rep1:
reads processed: 124657774
reads with at least one reported alignment: 84527594 (67.81%)
reads that failed to align: 10799459 (8.66%)
reads with alignments sampled due to -M: 29330721 (23.53%)

Input WT rep2:
reads processed: 109316591
reads with at least one reported alignment: 72907024 (66.69%)
reads that failed to align: 9982446 (9.13%)
reads with alignments sampled due to -M: 26427121 (24.17%)

H3K9me3 WT rep1:
reads processed: 90097243
reads with at least one reported alignment: 31794371 (35.29%)
reads that failed to align: 10177451 (11.30%)
reads with alignments sampled due to -M: 48125421 (53.41%)

H3K9me3 WT rep2:
reads processed: 79378787
reads with at least one reported alignment: 30203085 (38.05%)
reads that failed to align: 8440298 (10.63%)
reads with alignments sampled due to -M: 40735404 (51.32%)

Input KO rep1:
reads processed: 114898151
reads with at least one reported alignment: 75674261 (65.86%)
reads that failed to align: 9766797 (8.50%)
reads with alignments sampled due to -M: 29457093 (25.64%)

Input KO rep2:
reads processed: 134940877
reads with at least one reported alignment: 88494401 (65.58%)
reads that failed to align: 11547727 (8.56%)
reads with alignments sampled due to -M: 34898749 (25.86%)

H3K9me3 KO rep1:
reads processed: 96995449
reads with at least one reported alignment: 51447188 (53.04%)
reads that failed to align: 6973440 (7.19%)
reads with alignments sampled due to -M: 38574821 (39.77%)

H3K9me3 KO rep2:
reads processed: 88626502
reads with at least one reported alignment: 54718826 (61.74%)
reads that failed to align: 5658211 (6.38%)
reads with alignments sampled due to -M: 28249465 (31.87%)

H3 WT rep1:
reads processed: 118095580
reads with at least one reported alignment: 80695429 (68.33%)
reads that failed to align: 7844690 (6.64%)
reads with alignments sampled due to -M: 29555461 (25.03%)

H3 WT rep2:
reads processed: 104532754
reads with at least one reported alignment: 72769699 (69.61%)
reads that failed to align: 6665907 (6.38%)
reads with alignments sampled due to -M: 25097148 (24.01%)

H3 KO rep1:
reads processed: 120277067
reads with at least one reported alignment: 84264087 (70.06%)
reads that failed to align: 6955340 (5.78%)
reads with alignments sampled due to -M: 29057640 (24.16%)

H3 KO rep2:
reads processed: 100070101
reads with at least one reported alignment: 70669365 (70.62%)
reads that failed to align: 5779195 (5.78%)
reads with alignments sampled due to -M: 23621541 (23.60%)

IgG WT rep1:
reads processed: 99032939
reads with at least one reported alignment: 70525925 (71.21%)
reads that failed to align: 6275071 (6.34%)
reads with alignments sampled due to -M: 22231943 (22.45%)

IgG WT rep2:
reads processed: 65533816
reads with at least one reported alignment: 46164391 (70.44%)
reads that failed to align: 4256011 (6.49%)

	# reads with alignments sampled due to -M: 15113414 (23.06%)<br><br>IgG KO rep1:<br># reads processed: 101180925<br># reads with at least one reported alignment: 71908662 (71.07%)<br># reads that failed to align: 5461875 (5.40%)<br># reads with alignments sampled due to -M: 23810388 (23.53%)<br><br>IgG KO rep2:<br># reads processed: 89592105<br># reads with at least one reported alignment: 64125436 (71.57%)<br># reads that failed to align: 4887361 (5.46%)<br># reads with alignments sampled due to -M: 20579308 (22.97%)
Antibodies	Anti-H3K9me3 antibody (Abcam, ab8898, lot: GR148830-2)
Peak calling parameters	Peak calling with MACS2 was performed as follows:<br>macs2 callpeak -t H3K9me3.bed -c <input>.bed --broad -g mm --broad-cutoff 0.1 -f BED -n <outputfile>
Data quality	We first called peaks for each replicate against Input, which resulted in the following numbers of peaks (above FDR 5%):<br>H3K9me3 in WT rep1: 75,216<br>H3K9me3 in WT rep2: 72,062<br>H3K9me3 in KO rep1: 37,879<br>H3K9me3 in KO rep2: 81,813<br>Based on these, we composed a consensus dataset of 13,113 peaks that are present in WT but disappear in KO
Software	Alignment to mm9 mouse genome was performed with Bowtie (bowtie -t -v 2 -p 8 -M 1 -X 1000 --solexa-quals mm9)<br>Manipulations with BED files were performed using bedTools (https://github.com/arq5x/bedtools2).<br>Peaks were called with MACS2 (https://pypi.org/project/MACS2/) as detailed above.<br>HND domains were predicted with ChromHL v1.0.1 (https://github.com/TeifLab/ChromHL) DOI: 10.5281/zenodo.5987716
